# IRTKS negatively regulates antiviral immunity through PCBP2 sumoylation-mediated MAVS degradation

**DOI:** 10.1038/ncomms9132

**Published:** 2015-09-08

**Authors:** Pengyan Xia, Shuo Wang, Zhen Xiong, Buqing Ye, Li-Yu Huang, Ze-Guang Han, Zusen Fan

**Affiliations:** 1 Key Laboratory of Infection and Immunity of CAS, Institute of Biophysics, Chinese Academy of Sciences, 15 Datun Road, Chaoyang District, Beijing 100101, China; 2 University of Chinese Academy of Sciences, No.19A Yuquan Road, Beijing 100049, China; 3 Key Laboratory of Systems Biomedicine (Ministry of Education) and Collaborative Innovation Center of Systems Biomedicine, Shanghai Center for Systems Biomedicine, Shanghai Jiao Tong University, Shanghai 200240, China; 4 Shanghai-MOST Key Laboratory for Disease and Health Genomics, Chinese National Human Genome Center at Shanghai, 351 Guo Shou-Jing Road, Shanghai 201203, China

## Abstract

RNA virus infection is recognized by the RIG-I family of receptors that activate the mitochondrial adaptor MAVS, leading to the clearance of viruses. Antiviral signalling activation requires strict modulation to avoid damage to the host from exacerbated inflammation. Insulin receptor tyrosine kinase substrate (IRTKS) participates in actin bundling and insulin signalling and its deficiency causes insulin resistance. However, whether IRTKS is involved in the regulation of innate immunity remains elusive. Here we show that IRTKS deficiency causes enhanced innate immune responses against RNA viruses. IRTKS-mediated suppression of antiviral responses depends on the RIG-I-MAVS signalling pathway. IRTKS recruits the E2 ligase Ubc9 to sumoylate PCBP2 in the nucleus, which causes its cytoplasmic translocation during viral infection. The sumoylated PCBP2 associates with MAVS to initiate its degradation, leading to downregulation of antiviral responses. Thus, IRTKS functions as a negative modulator of excessive inflammation.

## Abstract (web summary)

RIG-I-MAVS signalling pathway is involved in mediating antiviral response to RNA viruses. Here the authors report that insulin receptor tyrosine kinase substrate (IRTKS) mediates MAVS degradation and thus acts as a negative regulator of the innate antiviral response.

## Graphical abstract

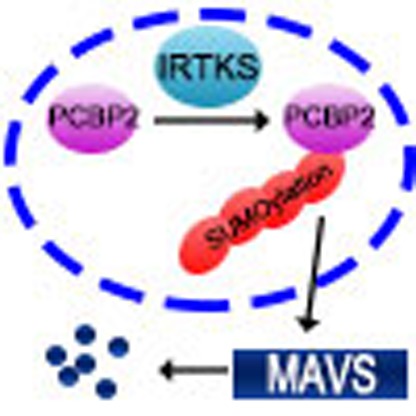

Innate immunity is the first defence line of a host against invading microbes^1^,^2^. The innate immune system utilizes germline-encoded pattern recognition receptors (PRRs), including membrane-bound Toll-like receptors and cytosolic retinoic acid-inducible gene-I (RIG-I)-like receptors (RLRs), to detect foreign pathogen invasion. Pathogen-associated molecular patterns derived from bacterial and viral elements are recognized by host PRRs^3^. All RLRs harbour a DEAD/H-box RNA helicase domain that binds to dsRNA. RIG-I and MDA5, sensors of RNA virus infection, contain an N-terminal tandem CARD domain that is critical to initiate type I interferon (IFN) secretion^4^,^5^,^6^. Once binding to viral RNAs, RIG-I and MDA5 undergo conformational change to activate another CARD-containing adaptor protein, mitochondrial antiviral signalling protein (MAVS, also known as VISA, IPS-1 and CARDIF)^7^,^8^,^9^,^10^. Then MAVS activates IRF3 and NF-κB to produce type I IFNs and other cytokines^11^,^12^,^13^, leading to further activation of the adaptive immunity and restriction of the infection.

To avoid damage to the host from excessive inflammation, antiviral signalling requires tight regulation to adequately eradicate invading pathogens. Given that MAVS coordinates signals from two independent central PRRs, the cell employs various mechanisms to modulate MAVS, including protein–protein interactions, changes of mitochondrial dynamics and post-translational modifications^12^. Recently, several negative regulators for MAVS have been reported^14^,^15^,^16^,^17^. Among these negative regulators, the host RNA-binding protein PCBP2 (poly(rC) binding protein 2) was originally identified to be involved in host cell mRNA stability and translational regulation of cellular mRNAs^18^. During virus infection, PCBP2 can associate with MAVS that acts as a scaffold to enhance AIP4-mediated degradation of MAVS^17^. Endogenous PCBP2 primarily resides in the nucleus but relocalizes to the cytoplasm where it initiates MAVS degradation on viral infection. However, the underlying mechanism by which PCBP2 undergoes nuclear export during virus infection is still unknown.

Insulin receptor tyrosine kinase substrate (IRTKS), a member of the IRSp53/MIM family, plays a pivotal role in the formation of plasma membrane protrusions^19^. It has been reported that IRTKS directly mediates the clustering of actin filaments^20^, and initiates pathogen-driven actin pedestal formation on bacterial infection^21^,^22^. In addition, IRTKS can be phosphorylated in response to insulin stimulation^20^. Moreover, IRTKS deficiency causes insulin resistance^23^. Insulin resistance might be implicated in clinical infections and immune regulations. However, whether IRTKS is involved in the regulation of innate immunity remains elusive. Here we show that IRTKS deficiency augments innate immune responses against RNA viruses. IRTKS mediates sumoylation of PCBP2 to cause its nuclear export on RNA virus infection, leading to MAVS degradation.

## Results

### IRTKS deficiency enhances anti-RNA virus activity

**Figure 1:**
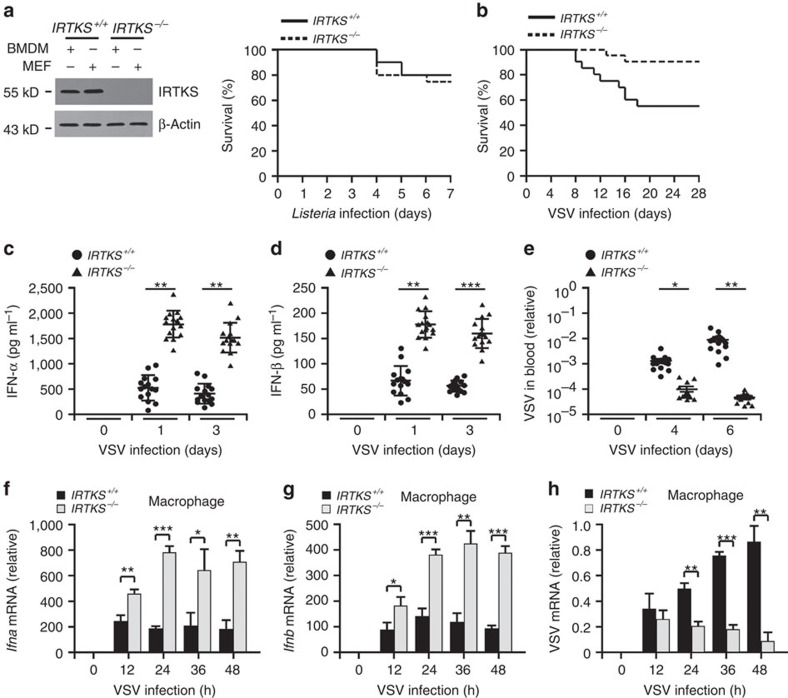
IRTKS deficiency enhances innate immune responses against RNA viruses. (**a**) IRTKS knockout mice display normal antibacterial activity. BMDMs and MEFs were subjected to immunoblotting with the indicated antibodies (left panel). *IRTKS*^*+/+*^ and *IRTKS*^−/−^ mice were intraperitoneally administrated with *Listeria* (2 × 10^6^) and survived mice were calculated in the following 7 days (right panel). *n*=20 mice per group. (**b**) *IRTKS*^*+/+*^ and *IRTKS*^−/−^ mice were intranasally inoculated with VSV (5 × 10^5^ p.f.u. for each mouse). Survival curves were calculated over infection. *n*=20. (**c**,**d**) *IRTKS*^*+/+*^ and *IRTKS*^−/−^ mice were intranasally inoculated with VSV (5 × 10^5^ p.f.u. for each mouse), followed by detection of serum interferons via an ELISA analysis. *n*=15. (**e**) Blood from mice treated as in **b** were collected and subjected to RNA extraction, followed by RT–PCR analysis with virus-specific primers. Virus values were normalized to that of β-actin. *n*=15. (**f**–**h**) *IRTKS*^*+/+*^ and *IRTKS*^−/−^ mice were intranasally inoculated with VSV (5 × 10^5^ p.f.u. for each mouse). IFN expression levels of peritoneal macrophages were examined at the indicated times (**f** and **g**). VSV mRNA from peritoneal macrophages was analysed through RT–PCR (**h**). For **f**–**h**, *n*=11. Data are shown as means±s.d. For (**c**–**e**), a two-way analysis of variance *post hoc* Bonferroni test was used; for **f**–**h**, a two-tailed unpaired Student's *t*-test was used. *P<0.05; **P<0.01; ***P<0.001. Data are representative of at least three independent experiments. p.f.u., plaque-forming unit.

We previously generated IRTKS knockout mice and found that IRTKS deletion exhibits insulin resistance^23^. Given that insulin resistance might be implicated in clinical infections, we then wanted to explore whether IRTKS was involved in the antimicrobial response. First, IRTKS was successfully deleted in bone marrow-derived macrophages (BMDMs) and mouse embryonic fibroblasts (MEFs) of *IRTKS*^*−/−*^ mice (Fig. 1a, left panel). We observed that the survival rate of *IRTKS*^*−/−*^ mice was similar to that of littermate control mice on infection with *Listeria monocytogenes* (Fig. 1a, right panel).

We next wanted to test whether IRTKS deficiency influenced the host's antiviral activity. We infected *IRTKS*^*−/−*^ mice with several RNA and DNA virus strains. Interestingly, *IRTKS*^*−/−*^ mice were more resistant to RNA virus vesicular stomatitis virus (VSV) infection compared with littermate *IRTKS*^*+/+*^ mice (Fig. 1b). However, when treated with DNA viruses such as herpes simplex virus (HSV), survival rates were similar between *IRTKS*^*+/+*^ and *IRTKS*^*−/−*^ mice (Supplementary Fig. 1a). These data indicate that *IRTKS*^*−/−*^ mice display enhanced antiviral activity against RNA viruses.

Type I IFNs are major cytokines to induce antiviral responses in the host^1^,^24^. With VSV infection, *IRTKS*^*−/−*^ mice expectedly produced higher levels of type I IFNs in sera than their littermate control mice (Fig. 1c,d). Consistently, viral titres were markedly declined in various tissues of *IRTKS*^*−/−*^ mice compared with *IRTKS*^*+/+*^ mice (Fig. 1e and Supplementary Fig. 1b,c). To further confirm the IRTKS deficiency-enhanced immune response against RNA viruses, we assayed IFN levels in *IRTKS*^*−/−*^ macrophages post viral stimulation. As expected, *IRTKS*^*−/−*^ macrophages generated higher levels of type I IFNs than those of their littermate control mice (Fig. 1f,g). Consequently, VSV replication was remarkably suppressed in *IRTKS*^*−/−*^ macrophages (Fig. 1h). Similarly, *IRTKS*^*−/−*^macrophages also produced higher levels of type I IFNs on Sendai virus (SeV) challenge (Supplementary Fig. 1d,e) and *IRTKS*^*−/−*^ mice were resistant to SeV infection (Supplementary Fig. 1f). By contrast, *IRTKS* deletion displayed no significant antiviral change compared with wild-type (WT) mice during DNA virus infection (Supplementary Fig. 1g–l). Taken together, IRTKS functions as an inhibitory modulator in the clearance of RNA viruses.

### IRTKS suppresses antiviral immunity via RIG-I-MAVS

**Figure 2:**
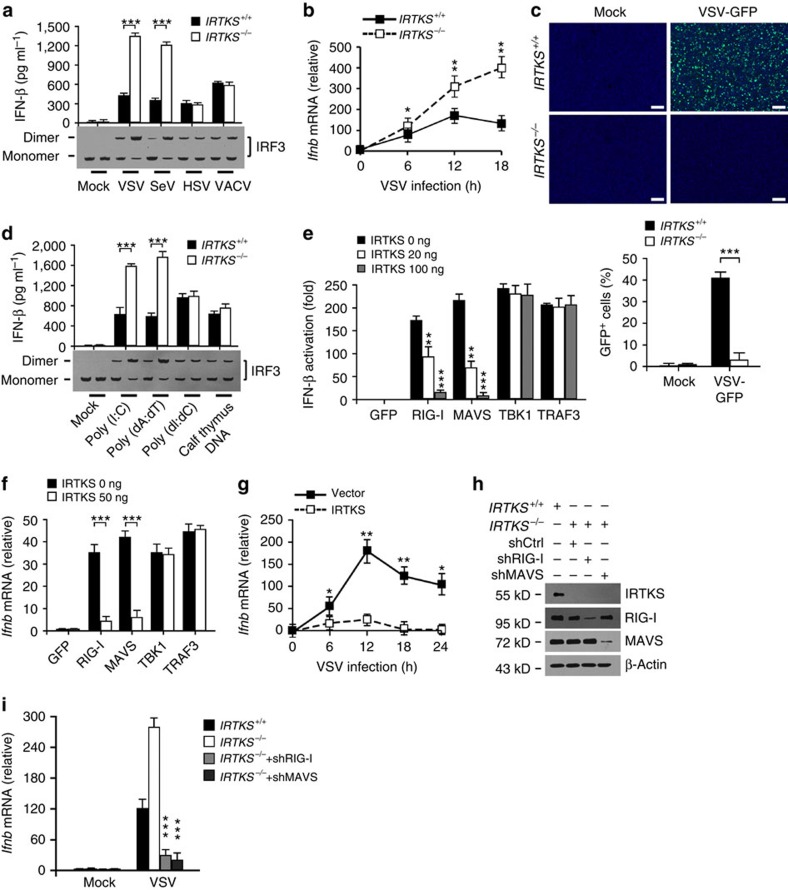
IRTKS-mediated suppression of antiviral immunity is RIG-I-MAVS dependent. (**a**) *IRTKS*^*+/+*^ and *IRTKS*^−/−^ BMDMs were infected with the indicated viruses (m.o.i.=5) for 18 h. IFN levels were assayed through ELISA (upper panel) and IRF3 dimerization was examined by immunoblotting (lower panel). (**b**) *IRTKS*^*+/+*^ and *IRTKS*^−/−^ BMDMs were infected with VSV (m.o.i.=5) for the indicated times, followed by RNA extraction and RT–PCR analysis of *Ifnb*. (**c**) *IRTKS*^*+/+*^ and *IRTKS*^−/−^ BMDMs were infected with VSV-GFP (m.o.i.=5) for 24 h, followed by examination with confocal microscopy (upper panel). Cells were counterstained with DAPI for nucleus. GFP-positive cells were calculated (lower panel). Scale bar, 200 μm. (**d**) *IRTKS*^*+/+*^ and *IRTKS*^−/−^ BMDMs were transfected with the indicated types of DNA or RNA (1 mg ml^−1^) for 18 h and analysed as **a**. (**e**) WT MEFs were transfected with IFN-β luciferase plasmid, various amounts of IRTKS and the indicated cDNAs involved in RNA sensing or GFP control for 24 h, followed by the analysis of promoter activity. (**f**) WT BMDMs were transfected with various amounts of IRTKS and the indicated cDNAs involved in RNA sensing or GFP control for 24 h, followed by RNA extraction for *Ifnb* expression (normalized to β-actin). (**g**) WT BMDMs expressing empty vector or IRTKS were incubated with VSV (m.o.i.=5) for the indicated times, followed by analysis of *Ifnb* mRNA (normalized to β-actin). (**h**,**i**) *IRTKS*^−/−^ BMDMs with RIG-I or MAVS knockdown (**h**) were incubated with VSV (m.o.i.=5) for 12 h, followed by IFN examination through RT–PCR (**i**). Data are shown as means±s.d. For **a**,**c**,**d**–**f**,**i**), a two-tailed unpaired Student's *t*-test was used; for **b**,**g**, a two-way analysis of variance *post hoc* Bonferroni test was used. *P<0.05; **P<0.01; ***P<0.001. Data represent at least three separate experiments. m.o.i., multiplicity of infection.

The induction of type I IFNs is modulated by transcription factors, including NF-κB and the IFN regulatory factors IRF3 and IRF7 (ref. 1). Activation of IRF3 and IRF7 needs phosphorylation by two IKK-related kinases, TBK1 and IKK^25^. On phosphorylation, these two IRFs undergo homodimerization and translocate into the nucleus, leading to the transcription of type I IFN genes. We then examined the activation of IRF3 in *IRTKS*^*−/−*^ BMDMs during virus challenge. As expected, RNA viruses were able to initiate enhanced activation of IRF3 in *IRTKS*^*−/−*^ macrophages and produced elevated levels of IFNs in these cells (Fig. 2a). However, DNA viruses had no such activity (Fig. 2a). In addition, IRTKS knockout sustained high levels of IFN expression after VSV infection (Fig. 2b). By contrast, for *IRTKS*^*+/+*^ cells, *Ifnb* mRNA levels were increased at early time points, whereas decreased at late time points post VSV challenge (Fig. 2b). Consequently, the replication of VSV in *IRTKS*^*−/−*^ cells was remarkably suppressed (Fig. 2c). However, IRTKS deficiency did not affect the IFN expression with HSV infection (Supplementary Fig. 2a) or the replication of HSV in these cells (Supplementary Fig. 2b). Since poly(dA:dT) can be converted to 5′-ppp RNA by RNA polymerase III (ref. 26), we then utilized poly(dA:dT) and poly(I:C) to mimic RNA virus infection. Similarly, poly(I:C) and poly(dA:dT) triggered robust activation of IRF3 and produced elevated levels of IFNs in *IRTKS*^*−/−*^ macrophages (Fig. 2d). By contrast, poly(dI:dC) and calf thymus DNA, mimicking viral DNAs, did not promote enhanced IRF3 transcription in *IRTKS*^*−/−*^ macrophages.

On RNA virus infection, RIG-I and MDA5 activate MAVS through the CARD–CARD interaction^1^,^27^. MAVS in turn triggers the activation of NF-κB and IRF3, suggesting the RIG-I-MAVS pathway is major means to clear RNA viruses. To determine whether IRTKS-mediated suppression of innate immunity depends on the RIG-I-MAVS signalling, we co-transfected MEF cells with increasing amounts of IRTKS plus members of the RIG-I-MAVS pathway. We found that IRTKS overexpression markedly inhibited IFN-β production induced by RIG-I and MAVS, but not by TBK1 or TRAF3 (Fig. 2e). However, IRTKS overexpression showed inhibitory effect on MDA5-mediated *Ifnb* expression (Supplementary Fig. 2c), but it did not block *Ifnb* expression even with a highest dose of IRTKS transfection during the period of VSV infection. These data are in agreement with a previous report showing that MDA5 has a distinct function from RIG-I in the innate immune response^28^,^29^. Moreover, IRTKS significantly suppressed endogenous *Ifnb* expression elicited by RIG-I and MAVS, but not by TBK1 or TRAF3 in macrophages (Fig. 2f). In addition, IRTKS overexpression remarkably inhibited *Ifnb* expression in macrophages with VSV infection (Fig. 2g). Similar observations were also achieved in poly(I:C)- or poly(dA:dT)-treated cells, but not in poly(dI:dC)- or calf thymus-DNA-treated cells (Supplementary Fig. 2d).

We next knocked down RIG-I or MAVS through shRNAs in *IRTKS*-deficient BMDMs (Fig. 2h). We noticed that RIG-I or MAVS knockdown almost blocked *Ifnb* expression in IRTKS-deficient cells (Fig. 2i). Altogether, IRTKS-mediated suppression of antiviral response relies on the RIG-I-MAVS signalling pathway.

### IRTKS interacts with PCBP2

**Figure 3:**
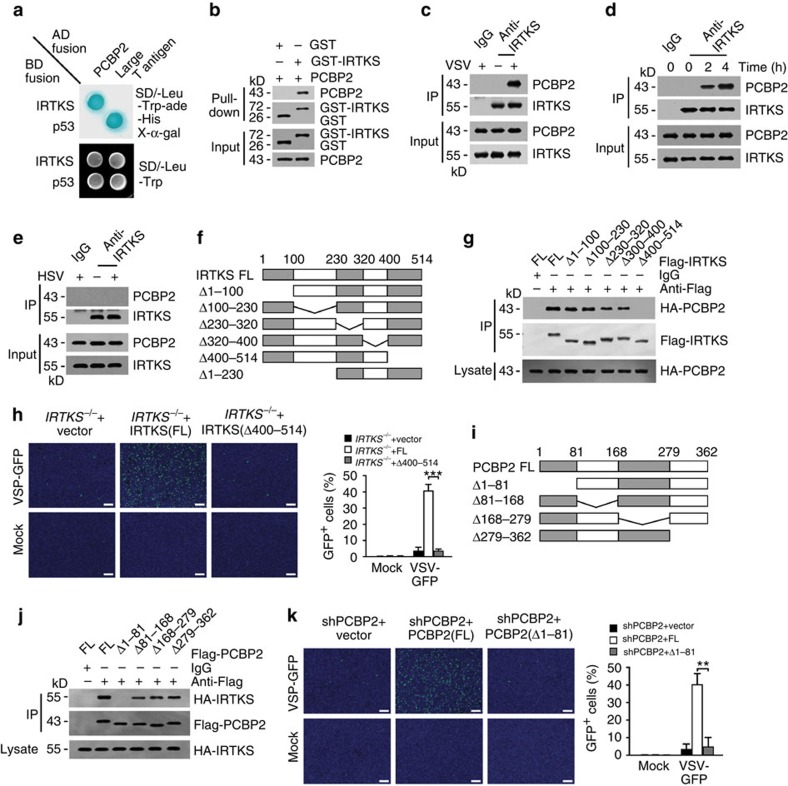
IRTKS associates with PCBP2. (**a**) IRTKS interacts with PCBP2 by yeast two-hybrid screening. Yeast strain AH109 was co-transfected with Gal4 DNA-binding domain (BD)-fused IRTKS and Gal4 activating domain (AD)-fused PCBP2. p53 and large T antigen were introduced as a positive control. (**b**) Recombinant GST-IRTKS and PCBP2 were subjected to GST pull-down assay. (**c**) WT BMDMS were infected with VSV (m.o.i.=5) for 2 h, followed by immunoprecipitation (IP) with anti-IRTKS antibody. (**d**) *IRTKS*^*+/+*^ and *IRTKS*^−/−^ mice were intranasally inoculated with VSV (5 × 10^5^ p.f.u. for each mouse) for the indicated times. Peritoneal macrophages were collected and lysed for IP with anti-IRTKS antibody. (**e**) WT BMDMS were infected with HSV (m.o.i.=5) for 2 h, followed by IP with anti-IRTKS antibody. (**f**) Scheme for IRTKS truncations. (**g**) The indicated Flag-tagged IRTKS truncations were co-transfected with FL HA-tagged PCBP2 into MEF cells, followed by IP with anti-Flag antibody. (**h**) *IRTKS*^−/−^ BMDMs were rescued with FL- or Δ400–514-IRTKS, followed by infection with VSV-GFP (m.o.i.=5) for 24 h. Cells were counterstained with DAPI (left panel). GFP-positive cells were calculated (right panel). Scale bar, 200 μm. (**i**,**j**) The indicated Flag-tagged PCBP2 truncations (**i**) were co-transfected with FL HA-tagged IRTKS into MEF cells, followed by IP with anti-Flag antibody (**j**). (**k**) PCBP2-silenced BMDMs were rescued with FL- or Δ1–81-PCBP2, followed by infection with VSV-GFP (m.o.i.=5) for 24 h. Cells were counterstained with DAPI (left panel). GFP-positive cells were calculated (right panel). Scale bar, 200 μm. Data are shown as means±s.d. A two-tailed unpaired Student's *t*-test was used. **P<0.01; ***P<0.001. Data are representative of at least three independent experiments. m.o.i., multiplicity of infection; p.f.u., plaque-forming unit.

To elucidate the IRTKS-mediated suppression of antiviral response, we screened a mouse cDNA library derived from murine bone marrow using IRTKS as a bait through a yeast two-hybrid approach. Seven positive clones were identified to be PCBP2, a negative regulator of MAVS^17^. Their interaction of IRTKS with PCBP2 was verified by the yeast two-hybrid assay (Fig. 3a). Moreover, recombinant IRTKS also associated with PCBP2 through a glutathione *S*-transferase (GST)-based pull-down assay (Fig. 3b). Notably, the association of IRTKS with PCBP2 was only observed in cells infected with VSV virus (Fig. 3c). In addition, the interaction of IRTKS with PCBP2 was significantly enhanced over the process of VSV infection (Fig. 3d). However, DNA viruses such as HSV virus failed to mediate this interaction (Fig. 3e). These data indicate that RNA viruses induce the interaction of IRTKS with PCBP2 during virus infection.

Using truncated IRTKS fragments, we mapped IRTKS (amino acid (aa) 400–514) was necessary and sufficient for association with PCBP2 (Fig. 3f,g). Importantly, restoration with the deleted IRTKS (IRTKS(Δ400–514)) did not rescue the suppression of VSV infection in IRTKS-deficient macrophages (Fig. 3h). However, restoration of the full-length (FL) IRTKS (IRTKS-FL) displayed robust VSV infection. These data suggest that the aa 400–514 fragment is necessary for IRTKS-mediated suppression of virus infection. Similarly, using truncated PCBP2 fragments, we defined PCBP2 (aa 1–81) was necessary and sufficient for interaction with IRTKS (Fig. 3i,j). Consistently, restoration of the deletion of aa 1–81 of PCBP2 (PCBP2(Δ1–81)) failed to rescue the suppression of VSV infection in PCBP2-silenced macrophages (Fig. 3k). Overall, the interaction of IRTKS with PCBP2 plays a critical role in the suppression of RNA virus infection.

### IRTKS promotes PCBP2-mediated degradation of MAVS

**Figure 4:**
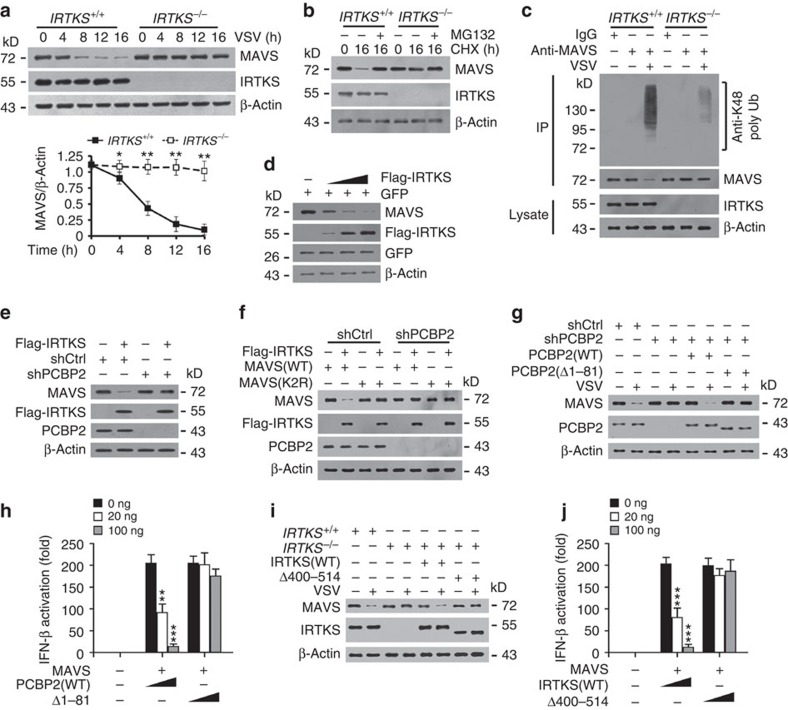
IRTKS promotes PCBP2-mediated degradation of MAVS. (**a**) *IRTKS*^*+/+*^ and *IRTKS*^−/−^ BMDMs were incubated with VSV (m.o.i.=5) for the indicated times, followed by immunoblotting with the indicated antibodies (upper panel). Ratios of MAVS/β-actin were calculated (lower panel). (**b**) *IRTKS*^*+/+*^ and *IRTKS*^−/−^ BMDMs were incubated with VSV (m.o.i.=5) for the indicated times in the presence of 20 μg ml^−1^ CHX and 10 μM MG132, followed by immunoblotting with the indicated antibodies. (**c**) *IRTKS*^*+/+*^ and *IRTKS*^−/−^ BMDMs were incubated with VSV (m.o.i.=5) for 16 h, followed by immunoprecipitation (IP) with anti-MAVS antibody. (**d**) Increasing amounts of Flag-tagged IRTKS and GFP control vector were co-transfected into BMDMs for 24 h. Protein levels were examined by immunoblotting with the indicated antibodies. (**e**) IRTKS promotes the degradation of MAVS through PCBP2. Flag-tagged IRTKS was transfected into scramble (shCtrl) or PCBP2-silenced BMDMs for 24 h, followed by immunoblotting with the indicated antibodies. (**f**) Flag-tagged IRTKS was co-transfected with WT MAVS or K2R-MAVS (K371R/K420R mutant) into shCtrl or PCBP2-silenced BMDMs for 24 h, followed by immunoblotting with the indicated antibodies. (**g**) PCBP2-depleted BMDMs were rescued with WT-PCBP2 or Δ1–81-PCBP2, followed by incubation with VSV (m.o.i.=5) for 16 h. Cell lysates were immunoblotted with the indicated antibodies. (**h**) WT MEFs were transfected with IFN-β luciferase plasmid, various amounts of PCBP2 and MAVS for 24 h, followed by analysis of promoter activity. (**i**) *IRTKS*^*+/+*^ and *IRTKS*^−/−^ BMDMs were rescued with WT-IRTKS or Δ400–514-IRTKS, followed by incubation with VSV (m.o.i.=5) for 16 h. (**j**) WT MEFs were transfected with IFN-β luciferase plasmid, various amounts of IRTKS and MAVS for 24 h. Data are shown as means±s.d. For **a**, a two-way analysis of variance (ANOVA) *post hoc* Bonferroni test was used; for **h**,**j**, a one-way ANOVA followed by a Dunnett *post hoc* test was used. *P<0.05; **P<0.01; ***P<0.001. Data are representative of at least three independent experiments. m.o.i., multiplicity of infection.

PCBP2 is known to augment MAVS degradation for immune regulation during RNA infection^17^. The association of IRTKS with PCBP2 prompted us to examine whether IRTKS regulated the protein stability of MAVS as well. As expected, IRTKS knockout prohibited the degradation of MAVS in BMDMs during VSV challenge (Fig. 4a). This degradation took place at the protein level, but not at the mRNA level (Supplementary Fig. 3a). Moreover, the proteasome inhibitor MG132 could block the degradation of MAVS (Fig. 4b), suggesting MAVS was degraded by the proteasome pathway. Consistently, K48-linked polyubiquitinated MAVS appeared in *IRTKS*^+/+^ BMDMs on VSV infection (Fig. 4c), whereas *IRTKS*^−/−^ cells displayed a very weak signal. More importantly, IRTKS overexpression facilitated MAVS degradation with a dose-dependent manner in macrophages on VSV challenge (Fig. 4d). However, IRTKS overexpression did not affect the mRNA stability of MAVS (Supplementary Fig. 3b). These results suggest that IRTKS enhances proteasome-mediated degradation of MAVS during virus infection.

Of note, IRTKS overexpression failed to induce MAVS degradation in PCBP2-depleted macrophages (Fig. 4e), suggesting that IRTKS-mediated suppression of antiviral response depends on PCBP2. It has been reported that PCBP2 plays a pivotal role in the degradation of MAVS^17^. They also demonstrated that Lys371 and Lys420 of MAVS are the two residues for K48-linked polyubiquitination. We then generated the degradation-resistant mutant K371R/K420R-MAVS and overexpressed it in macrophages. We observed that IRTKS did not induce the degradation of K371R/K420R-MAVS even in shCtrl-treated macrophages (Fig. 4f). However, IRTKS potentiated the degradation of WT MAVS only in the presence of PCBP2. We then rescued PCBP2(FL) or PCBP2(Δ1–81) in PCBP2-silenced BMDMs. PCBP2(Δ1–81) restoration failed to induce the degradation of MAVS post virus stimulation (Fig. 4g). Consequently, PCBP2(Δ1–81) restoration did not inhibit MAVS-mediated type I IFN generation (Fig. 4h). By contrast, PCBP2(FL) restoration remarkably prohibited type I IFN production. Finally, IRTKS(Δ400–514) restoration could not induce the degradation of MAVS on virus stimulation either (Fig. 4i). Consistently, IRTKS(Δ400–514) restoration did not suppress MAVS-mediated type I IFN production (Fig. 4j). Altogether, IRTKS potentiates the degradation of MAVS through interaction with PCBP2.

### IRTKS recruits Ubc9 to sumoylate PCBP2 on virus infection

**Figure 5:**
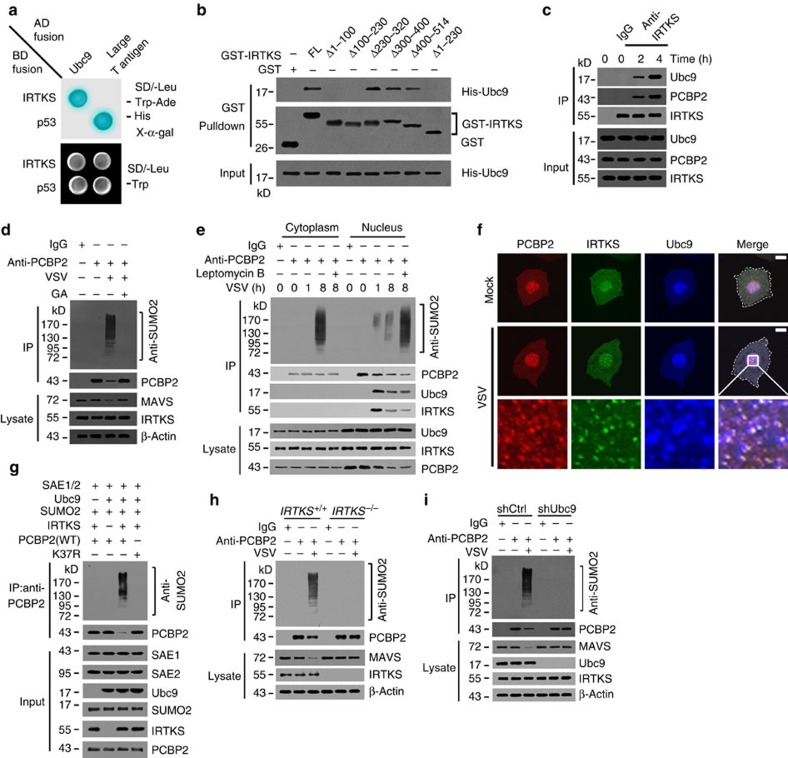
IRTKS recruits Ubc9 that sumoylates PCBP2 in the nucleus right after VSV infection. (**a**) Yeast strain AH109 was co-transfected with Gal4 DNA-binding domain (BD)-fused IRTKS and Gal4 activating domain (AD)-fused Ubc9. p53 and large T antigen were introduced as a positive control. (**b**) Mapping the interacting regions between IRTKS and Ubc9. The indicated GST-tagged IRTKS truncations were incubated with His-tagged Ubc9, followed by GST pull-down assay. (**c**) WT mice were intranasally inoculated with VSV (5 × 10^5^ p.f.u. for each mouse) for the indicated times. Peritoneal macrophages were collected and lysed for immunoprecipitation (IP) with anti-IRTKS antibody. Immunoprecipitates were immunoblotted with the indicated antibodies. (**d**) WT BMDMs were incubated with VSV (m.o.i.=5) for 8 h with or without ginkgolic acid (GA, 20 μm), followed by IP with anti-PCBP2 antibody. Immunoprecipitates were detected with anti-SUMO2 antibody. (**e**) WT BMDMs were incubated with VSV (m.o.i.=5) for the indicated times with or without 40 nM leptomycin B, followed by cytoplasmic and nuclear separation. Lysates were immunoprecipitated with anti-PCBP2 antibody, followed by immunoblotting with the indicated antibodies. (**f**) WT BMDMs were incubated with VSV (m.o.i.=5) for 1 h, followed by immunostaining with the indicated antibodies. Nuclei were counterstained with DAPI. Scale bar, 10 μm. (**g**) Recombinant PCBP2 was incubated with the indicated recombinant proteins in the presence of 5 mM ATP, followed by IP with antibody against PCBP2. The immunoprecipitates were immunoblotted with the indicated antibodies. (**h**) *IRTKS*^*+/+*^ and *IRTKS*^−/−^ BMDMs were incubated with VSV (m.o.i.=5) for 8 h, followed by IP with anti-PCBP2 antibody. Immunoprecipitates were detected with anti-SUMO2 antibody. (**i**) shCtrl- or Ubc9-silenced BMDMs were incubated with VSV (m.o.i.=5) for 8 h, followed by IP with anti-PCBP2 antibody. Data are shown as means±s.d. Data were repeated at least three times with similar results. m.o.i., multiplicity of infection; p.f.u., plaque-forming unit.

Among the positive clones screened out from the above yeast two-hybrid assay, 21 clones were identified to be Ubc9. The interaction of IRTKS with Ubc9 was validated by yeast two-hybrid assay (Fig. 5a). We then mapped the Ubc9-binding site on IRTKS. We noticed that the aa 1–230 fragment of IRTKS was necessary and sufficient for interacting with Ubc9 (Fig. 5b). More importantly, with VSV stimulation, anti-IRTKS antibody was able to precipitate Ubc9 and PCBP2 in macrophages (Fig. 5c), suggesting that IRTKS associated with Ubc9 and PCBP2 during virus challenge. Ubc9 is the only E2 ligase for protein sumoylation^30^,^31^. Then we examined whether PCBP2 or IRTKS underwent sumoylation during virus infection. Interestingly, we observed that PCBP2 was sumoylated on VSV challenge (Fig. 5d). Treatment with the sumoylation inhibitor ginkgolic acid^32^ blocked the sumoylation of PCBP2. During VSV infection, polysumoylated PCBP2 was further verified by immunoblotting with anti-PCBP2 or anti-small ubiquitin-like modifier 2 (anti-SUMO2) antibody (Supplementary Fig. 4a,b). Furthermore, PCBP2 sumoylation was caused by SUMO2, but not by SUMO1 (Supplementary Fig. 4c,d), which was validated by immunoblotting with anti-HA antibody. These data suggest that PCBP2 undergoes SUMO2-mediated polysumoylation during virus infection.

We next wanted to determine the cellular location in which PCBP2 was sumoylated on VSV infection. We infected BMDMs with VSV virus and performed fractionation of the cytoplasm and nucleus during early and late time points. At 1 h post VSV infection, PCBP2 sumoylation appeared in the nucleus, not detectable in the cytoplasm (Fig. 5e). However, at 8 h after VSV infection, PCBP2 sumoylation mainly existed in the cytoplasm (Fig. 5e), whereas only weak signals were picked in the nucleus. With treatment of the nuclear export inhibitor leptomycin B before VSV infection, PCBP2 sumoylation only appeared in the nucleus at 8 h, but no sumoylated signals in the cytoplasm (Fig. 5e). These data indicate that the PCBP2 sumoylation takes place in the nucleus during VSV infection. Notably, PCBP2 and Ubc9 were mainly localized in the nucleus in Mock-treated BMDMs (Fig. 5f). Whereas IRTKS distributed in the nucleus and cytoplasm. At early time post VSV infection, PCBP2, Ubc9 and IRTKS exhibited colocalization in the nucleus of BMDMs (Fig. 5f). Furthermore, VSV particles entered into the nucleus and colocalized with these proteins at an early stage of VSV challenge (Supplementary Fig. 4e).

We further verified our observations with an *in vitro* reconstitution assay. We found that PCBP2 could be sumoylated by Ubc9 with the *in vitro* assay (Fig. 5g). Lysine 37 was predicted to be the only sumoylation site on PCBP2 through a GPS-anti-small ubiquitin-like modifier (SUMO) predicting tool^33^. We generated several PCBP2 mutants carrying lysine to arginine mutations. As expected, the K37R mutation impaired the sumoylation of PCBP2 via the *in vitro* assay (Fig. 5g). More importantly, IRTKS was required for the sumoylation of PCBP2 (Fig. 5g). Without IRTKS, PCBP2 did not undergo sumoylation. Expectedly, *IRTKS* deficiency abrogated the sumoylation of PCBP2 with VSV challenge (Fig. 5h), which maintained the stability of MAVS. Finally, Ubc9 depletion also abolished the PCBP2 sumoylation (Fig. 5i). Taken together, IRTKS recruits Ubc9 to sumoylate PCBP2 in the nucleus on RNA virus infection.

### IRTKS promotes PCBP2 translocation and MAVS degradation

**Figure 6:**
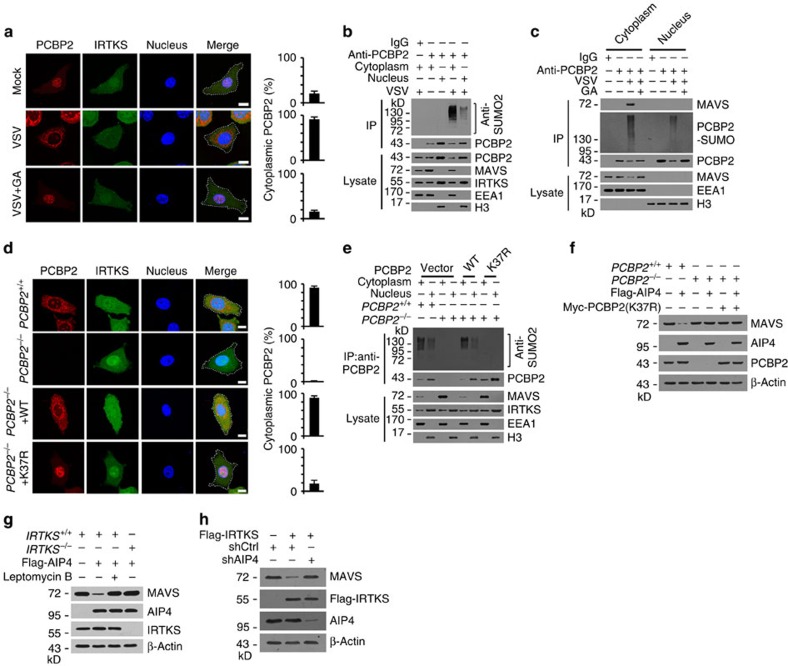
IRTKS-mediated PCBP2 sumoylation causes its cytoplasmic translocation leading to MAVS degradation. (**a**) *IRTKS*^*+/+*^ and *IRTKS*^−/−^ BMDMs were incubated with VSV (m.o.i.=5) for 8 h with or without ginkgolic acid (GA) (20 μm), followed by immunostaining with the indicated antibodies. Percentages of cytoplasmic PCBP2 (cytoplasmic PCBP2/total PCBP2) were calculated (right panel). At least 200 cells were counted. Scale bar, 10 μm. (**b**) WT BMDMs were incubated with VSV (m.o.i.=5) for 8 h, followed by immunoprecipitation (IP) with anti-PCBP2 antibody using cytoplasmic or nuclear fractions. (**c**) WT BMDMs were incubated with VSV (m.o.i.=5) for 8 h with or without GA (20 μm), followed by cytoplasmic and nuclear separation. IP was performed using anti-PCBP2 antibody. Immunoprecipitates were detected with the indicated antibodies. (**d**) *PCBP2*^*+/+*^ and *PCBP2*^−/−^ BMDMs rescued with WT or K37R-PCBP2 were incubated with VSV (m.o.i.=5) for 8 h, followed by immunostaining with the indicated antibodies. Percentages of cytoplasmic PCBP2 (cytoplasmic PCBP2/total PCBP2) were calculated (right panel). At least 200 cells were counted. Scale bar, 10 μm. (**e**) *PCBP2*^*+/+*^ and *PCBP2*^−/−^ BMDMs rescued with the indicated PCBP2 plasmids were incubated with VSV (m.o.i.=5) for 8 h, followed by IP with anti-PCBP2. (**f**) *PCBP2*^*+/+*^ and *PCBP2*^−/−^ MEFs were transfected with Flag-AIP4 and Myc-PCBP2 (K37R) for 24 h, followed by immunoblotting with the indicated antibodies. (**g**) *IRTKS*^*+/+*^ and *IRTKS*^−/−^ MEFs were transfected with Flag-AIP4 for 24 h with or without 40 nM leptomycin B, followed by immunoblotting with the indicated antibodies. (**h**) shCtrl- or AIP4-silenced MEFs were transfected with Flag-IRTKS for 24 h, followed by immunoblotting with the indicated antibodies. Data are shown as means±s.d. Data are representative of at least three separate experiments. m.o.i., multiplicity of infection.

It has been reported that protein sumoylation is involved in the nuclear–cytoplasmic transport of various proteins^34^,^35^. Given that MAVS is a mitochondrial membrane adaptor, the interaction of PCBP2 with MAVS requires the nuclear export of PCBP2. Therefore, we next examined whether the sumoylation of PCBP2 triggered its cytoplasmic translocation. Expectedly, VSV stimulation caused cytoplasmic translocation of PCBP2 post 8-h infection (Fig. 6a). Moreover, the sumoylation inhibitor ginkgolic acid blocked the nuclear export of PCBP2 with VSV challenge (Fig. 6a). As shown in the Fig. 5e, leptomycin B also blocked cytoplasmic translocation of sumoylated PCBP2 after VSV challenge. Consistently, a large amount of sumoylated PCBP2 appeared in the cytoplasm of macrophages post 8 h of VSV stimulation (Fig. 6b), whereas only a little amount of sumoylated PCBP2 remained in the nucleus. Accordingly, MAVS underwent degradation in the cytoplasm of VSV-infected macrophages (Fig. 6b). These observations were further validated by cellular fractionation (Fig. 6c). In addition, sumoylated PCBP2 neither affect the interaction between MAVS and PCBP2, nor the association of RIG-I or MDA5 (Supplementary Fig. 5a).These data indicate that the sumoylated PCBP2 leads to its nuclear export during the progress of virus infection.

We next deleted PCBP2 in macrophages via a CRISPR/Cas9 approach (Supplementary Fig. 5b–d). We rescued WT-PCBP2 or K37R-PCBP2 in PCBP2-deleted macrophages. We observed that WT-PCBP2 restoration was able to cause the nuclear export of PCBP2 after VSV infection (Fig. 6d). However, the K37R-PCBP2 mutant abolished such activity. Consistently, K37R-PCBP2 restoration impaired the sumoylation of PCBP2 (Fig. 6e), which kept MAVS stability as that of empty vector-treated control cells. By contrast, WT-PCBP2 restoration still induced the sumoylation of PCBP2 and caused the cytoplasmic translocation of PCBP2 (Fig. 6e), leading to degradation of MAVS. AIP4 has been reported to mediate MAVS ubiquitination in a PCBP2-dependent manner^16^. We noticed that PCBP2 sumoylation is required for AIP4 activation (Fig. 6f). Cytoplasmic translocation of PCBP2 and IRTKS are also needed for MAVS degradation (Fig. 6g). IRTKS-mediated MAVS degradation is AIP4 dependent (Fig. 6h).

**Figure 7:**
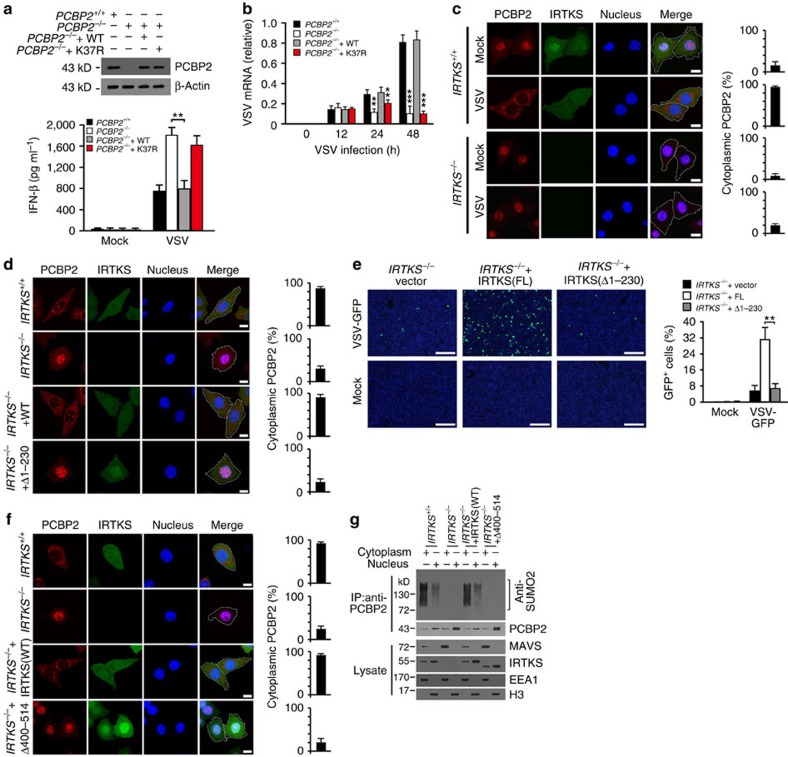
IRTKS-mediated PCBP2 sumoylation is required for MAVS negative regulation during RNA virus infection. (**a**) *PCBP2*^−/−^ BMDMs were transfected with WT- or K37R-PCBP2 for 18 h (upper panel), followed by infection with VSV (m.o.i.=5) for 18 h. IFN levels were detected by ELISA (lower panel). (**b**) *PCBP2*^−/−^ BMDMs were transfected with WT- or K37R-PCBP2 for 18 h, followed by infection with VSV (m.o.i.=5) for the indicated times. VSV mRNA of BMDMs was analysed by RT–PCR. (**c**) *IRTKS*^*+/+*^ and *IRTKS*^−/−^ BMDMs were incubated with VSV (m.o.i.=5) for 8 h, followed by immunostaining with the indicated antibodies. Percentages of cytoplasmic PCBP2 (cytoplasmic PCBP2/total PCBP2) were calculated (right panel). At least 200 cells were counted. Scale bar, 10 μm. (**d**) *IRTKS*^*+/+*^ and *IRTKS*^−/−^ BMDMs rescued with WT or Δ1–230-IRTKS were incubated with VSV (m.o.i.=5) for 8 h, followed by immunostaining with the indicated antibodies. Percentages of cytoplasmic PCBP2 (cytoplasmic PCBP2/total PCBP2) were calculated (right panel). At least 200 cells were counted. Scale bar, 10 μm. (**e**) *IRTKS*^*+/+*^ and *IRTKS*^−/−^ BMDMs were rescued with WT or Δ1–230-IRTKS, followed by infection with VSV-GFP (m.o.i.=5) for 24 h. Cells were counterstained with DAPI (left panel). GFP-positive cells were calculated (right panel). Scale bar, 200 μm. (**f**) *IRTKS*^*+/+*^ and *IRTKS*^−/−^ BMDMs rescued with WT or Δ400–514-IRTKS were incubated with VSV (m.o.i.=5) for 8 h, followed by immunostaining with the indicated antibodies. Percentages of cytoplasmic PCBP2 (cytoplasmic PCBP2/total PCBP2) were calculated (right panel). At least 200 cells were counted. Scale bar, 10 μm. (**g**) *IRTKS*^*+/+*^ and *IRTKS*^−/−^ BMDMs rescued with the indicated IRTKS plasmids were incubated with VSV (m.o.i.=5) for 8 h, followed by immunoprecipitation (IP) with anti-PCBP2 antibody using cytoplasmic or nuclear fractions. Immunoprecipitates or cell lysates were immunoblotted with the indicated antibodies. Data are shown as means±s.d. For **a**,**e**, a two-tailed unpaired Student's *t*-testwas used; for **b**, a one-way analysis of variance followed by a Dunnett *post hoc* test was used using *PCBP2*^*+/+*^ cells as controls.*P<0.05; **P<0.01; ***P<0.001. Data are representative of at least three separate experiments. m.o.i., multiplicity of infection.

Notably, PCBP2 knockout markedly produced type I IFNs (Fig. 7a). However, K37R-PCBP2 restoration did not suppress the production of IFN-β in PCBP2-deleted macrophages (Fig. 7a), while WT-PCBP2 restoration inhibited the generation of IFN-β. Consequently, K37R-PCBP2 restoration still repressed the virus titres and replication rate as *PCBP2*^+/+^ cells (Fig. 7b and Supplementary Fig. 5e). These data indicate that the sumoylation of PCBP2 is involved in the regulation of MAVS-mediated virus clearance.

Notably, *IRTKS* deletion abrogated the nuclear export of PCBP2 after 8 h of VSV challenge (Fig. 7c), suggesting that IRTKS played a critical role in the cytoplasmic translocation of PCBP2. As shown in Fig. 5b, the aa 1–230 fragment of IRTKS is necessary for association with Ubc9. To further confirm the critical role of IRTKS in PCBP2 sumoylation, we then rescued WT-IRTKS or Δ1–230-IRTKS in *IRTKS*^*−/−*^ macrophages. We found that Δ1–230-IRTKS restoration did not cause the cytoplasmic translocation of PCBP2 after 8 h of VSV infection (Fig. 7d). Accordingly, Δ1–230-IRTKS restoration did not suppress antiviral activity in IRTKS-deficient cells either (Fig. 7e), suggesting a critical role of IRTKS in the sumoylation of PCBP2. Finally, Δ400–514-IRTKS restoration in *IRTKS*^*−/−*^ macrophages did not induce the nuclear export of PCBP2 with VSV challenge either (Fig. 7f). Consistently, Δ400–514-IRTKS restoration disrupted the PCBP2 sumoylation (Fig. 7g, upper panel), and still sustained the stability of MAVS (Fig. 7g, lower panel). Overall, IRTKS-mediated PCBP2 sumoylation is required for its cytoplasmic translocation that associates with MAVS, leading to MAVS degradation.

## Discussion

**Figure 8:**
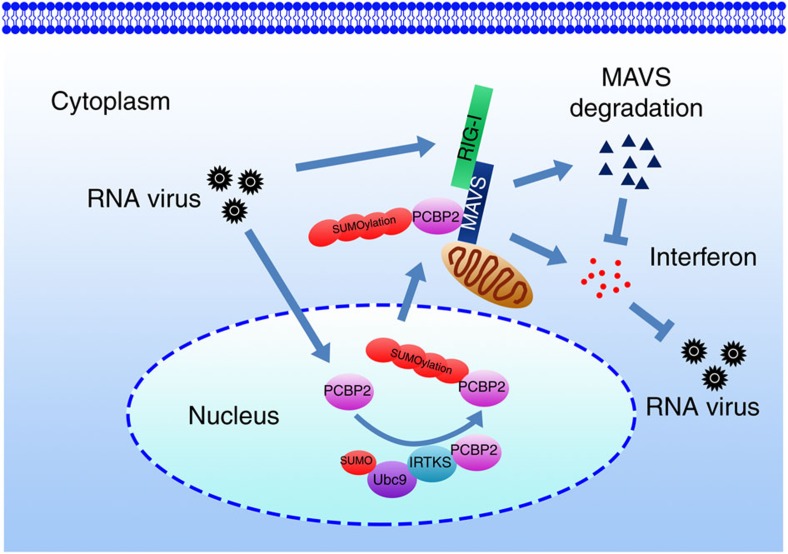
A work model for IRTKS-mediated MAVS degradation during RNA virus infection.

RNA virus infection is recognized by the RIG-I family of receptors to activate the mitochondrial adaptor protein MAVS, leading to clearance of viruses. MAVS, an innate immune signalling adaptor, integrates signals from two independent cytosolic PRRs to trigger activation of IKK and TBK, which consequently activate NF-κB and IRF3 to induce production of type I IFNs^1^. Antiviral signalling activation is an extremely powerful cellular response that requires strict modulation to adequately eradicate invading viruses while avoiding damage to the cell from overzealous inflammation. In this study, we define that IRTKS as an inhibitory modulator for the RIG-I-MAVS signalling pathway. IRTKS can recruit the E2 ligase Ubc9 to sumoylate PCBP2 in the nucleus that causes its cytoplasmic translocation. In the cytoplasm, the sumoylated PCBP2 associates with MAVS to initiate MAVS degradation, leading to downregulation of host immune response (Fig. 8).

On activation of the RLR signalling pathway, MAVS undergoes prion-like polymerization to form a MAVS signalosome^11^,^12^, resulting in activation of the cytosolic kinases IKK and TBK1. In this process, many factors of the host tightly regulate the RIG-I-MAVS signalling pathway to balance the virus infection-elicited inflammation. It has been reported that some viruses, including hepatitis C virus and enterovirues, employ their endogenous proteinases to directly cleave MAVS^8^,^36^,^37^, leading to the inactivation of the MAVS-mediated antiviral responses. However, most viruses are recognized by host PRRs to initiate cellular responses, which consequently regulate antiviral immunity. Post-translational modifications of MAVS and its associated partners are a main aspect of host cell modulation of antiviral responses. Several E3 ubiquitin ligases, including TNF125 (ref. 38), TRIM25 (ref. 39) and AIP4 (ref. 17), have been identified to ubiquitinate MAVS for its degradation. PCBP2 could be act as a physical scaffold to link MAVS with its E3 ligases for its ubiquitination^17^. Notably, PCBP2 resides mainly in the nucleus in a steady state. However, it is unknown how PCBP2 relocalizes to the cytosol where it associates with MAVS on virus infection. Herein, we show that IRTKS negatively regulates antiviral responses against RNA viruses in a RIG-I-MAVS-dependent manner. PCBP2 sumoylation mediated by IRTKS took place in the nucleus on RNA virus infection, which causes its cytoplasmic translocation to interact with MAVS during the late stage of infection, leading to MAVS degradation. PCBP2 sumoylation and IRTKS are required for AID4-mediated MAVS degradation. Moreover, MDA5 expression increases *Ifnb* expression only at late time of VSV infection, which is in agreement with recent reports^28^,^29^. These observations suggest that IRTKS might play a role at the early time points of the antiviral response that is RIG-I mediated. Therefore, the PCBP2 sumoylation plays a critical role in IRTKS-mediated suppression of antiviral activity against RNA viruses.

Protein sumoylation plays an important role in various cellular processes, including cell proliferation, apoptosis, transcription regulation and so on^40^,^41^. Like the ubiquitin system, the SUMO system targets a large number of proteins to fulfil numerous functions^42^,^43^. However, the salient difference from the ubiquitin system is the simplicity of the enzymatic apparatus for sumoylation and de-sumoylation. Up to date, only one SUMO-conjugating enzyme Ubc9 has been identified^41^. In this study, we demonstrate that IRTKS can recruit Ubc9 to sumoylate PCBP2 at Lys37 in the nucleus during the early stage of RNA virus infection. Over the process of RNA virus infection, sumoylated PCBP2 causes its cellular switch for nuclear versus cytoplasmic localization, where the sumoylated PCBP2 associates with MAVS to initiate its degradation. The sumoylation inhibitor or Ubc9 depletion abolishes the sumoylation of PCBP2, indicating that PCBP2 is a novel substrate for sumoylation during RNA virus infection.

In the inner membrane compartment, IRTKS regulates bundles of actin filaments^20^. During intracellular bacterial infection, bacteria utilize the cytoplasmic actin activators to generate pathogen-driven actin pedestal right after the bacterial invasion^22^. IRTKS can be directly activated by the tyrosine kinase Src through phosphorylation, leading to enhanced cell migration^44^. In addition, IRTKS can interact with the epidermal growth factor receptor to activate the ERK signalling, which is involved in the pathogenesis of hepatocellular carcinoma^45^. Our recent report showed that IRTKS is phosphorylated in response to insulin stimulation, whose deletion causes insulin resistance^23^. Insulin resistance might be implicated in clinical infections and immune regulations. Herein, we show that IRTKS deficiency does not influence the antibacterial response in mice. Quite interestingly, IRTKS-deficient mice exhibit elevated antiviral activities against RNA viruses, but not DNA viruses. However, the underlying mechanism of IRTKS-mediated immune suppression in response to distinct viruses needs to be further investigated.

IRTKS was originally cloned from endocrine organs^46^. As an insulin receptor tyrosine substrate, IRTKS is phosphorylated after activation of insulin signalling^20^. Insulin, as the primary anabolic hormone, modulates a variety of physiological processes, including growth, differentiation, apoptosis, as well as synthesis and breakdown of lipid, protein and glucose^47^. Insulin binds to its insulin receptor to activate the receptor intrinsic tyrosine kinases, leading to activation of the PI3K/Akt pathway^48^,^49^. Insulin signalling is indispensable for glucose metabolism in cells of the muscle and adipose tissues^50^. We previously demonstrated that IRTKS-deficient mice display hyperglycaemia, hyperinsulinaemia, glucose intolerance and decreased insulin sensitivity^23^, suggesting IRTKS is implicated in the pathogenesis of diabetes. The pathogenesis of diabetes might be closely related to excessive autoimmune responses^51^. However, the mechanism leading to autoimmunity in diabetes still remains elusive. Recent onset of type 1 diabetes is strongly correlated with infection by RNA viruses such as enteroviruses^52^,^53^. Moreover, a transcriptional signature of type I IFNs precedes islet autoimmunity^54^. Type I IFNs play a critical role in many autoimmune diseases via various immune modulatory actions^55^. We show that IRTKS targets the RIG-I-MAVS signalling axis that modulates the production of type I IFNs as a negative modulator in infection of RNA viruses. Furthermore, IRTKS-mediated immune suppression differs in response to different types of viruses. Thus, our findings will prompt us to further investigate the connection of IRTKS between the virus infections and the pathogenesis of autoimmune diseases.

## Methods

### Antibodies and reagents

Antibodies used were: anti-IRTKS and anti-EEA1 were from Santa Cruz Biotechnology; anti-IRF3, anti-MAVS, anti-K48-polyubiquitin, anti-H3 and anti-Ubc9 were from Cell Signaling Technology; anti-PCBP2 and anti-SUMO2 were from MBL International Corporation; anti-GST, anti-green fluorescent protein (GFP), anti-HA, anti-FLAG, anti-β-actin and anti-His antibodies were from Sigma-Aldrich; Donkey anti-rabbit or anti-mouse secondary antibodies conjugated with Alexa-488, Alexa-594 or Alexa-405 were purchased from Molecular Probes. HRP-conjugated secondary antibodies were from Santa Cruz Biotechnology. Paraformaldehyde, cycloheximide, MG132, ginkgolic acid, isopropyl-β-D-thiogalactopyranoside, 4,6-diamidino-2-phenylindole andpropidiumiodide were from Sigma-Aldrich.

### Cells and culture

For BMDMs, bone marrow cells were aspirated from mouse femurs, followed by culture in RPMI 1640 media containing 10% fetal bovine serum, 50 ng ml^−1^ macrophage colony stimulating factor (MCSF) for 7 days. For macrophage transfection, BMDMs (1 × 10^6^) were resuspended in 100 μl Nucleofector solution buffer (Lonza) containing 5 μg RNA or other substrates, followed by transfection using the Nucleofector Program Y-001 on Amaxa nucleofector II device (Lonza). Cells were recovered in RPMI 1640 media containing 4 mM L-glutamine, 1.5 g l^−1^ sodium bicarbonate and 10% heat-inactivated fetal bovine serum for 6 h, followed by flow cytometric sorting for viable cells.

### Animals and viruses

Mouse experiments complied with ethical regulations and were approved by the Institutional Animal Care and Use Committees at the Institute of Biophysics, Chinese Academy of Sciences. IRTKS mice were generated through embryo injection of embryonic stem cells with a complete deletion of exon 1 of IRTKS provide by Shanghai Bimodal Organism Science & Technology Development^23^. IRTKS knockout mice were backcrossed to C57BL/6 background for more than six generations. Female mice at an age of 12 weeks were used in this study. For preparation of viruses, viruses were incubated with Vero cells, followed by supernatant collection 48 h later. SeV viruses were cultured in embryonated eggs. Supernatants were ultra-centrifuged at 25,000*g* for 2 h. Pellets were resuspended in RPMI 1640. For virus infection *in vivo*, mice were either intravenously injected with viruses or intranasally inoculated with viruses for long time observation.

### Plasmid construction

SAE1, SAE2, Ubc9, SUMO2, IRTKS, PCBP2, RIG-I and MDA5 were cloned from a murine bone marrow library^29^. MAVS (also known as VISA) was a gift from Hongbing Shu (Wuhan University). SAE1, SAE2, Ubc9 andSUMO2 were subcloned into pET-28a vectors for His-tagged protein expression.PCBP2 and IRTKS were subcloned into pFlag-CMV2 or pCMV-HA for expression in mammalian cells. PCBP2 and IRTKS were also subcloned into pGEX-6p-1 for GST-fusion protein expression in *Escherichia coli*.

### ELISA-based interferon determination

Supernatants from cultured cells or sera were collected at the indicated times. Cytokines were analysed by ELISA kits (R&D Systems) with the manufacturer's instructions.

### Luciferase assay

IFN-β luciferase reporter was constructed as described^26^. Luciferase reporter vectors were co-transfected with pRL-TK (as an internal control reporter vector) into MEF cells by electroporation. Luciferase assays were performed with guidelines provided by the manufacturer (Promega).

### Yeast two-hybrid screening

Yeast two-hybrid screening was performed as described previously^56^. Briefly, IRTKS was cloned into pGBKT7 vector (BD-IRTKS). Yeast AH109 cells were transfected with BD-IRTKS and plasmids containing a mouse bone marrow cDNA library (Clontech/Takara) and then plated on selective synthetic defined (SD) media. Selected clones were isolated for DNA sequencing. X-α-gal assay was carried out by the manufacturer's instructions.

### Immunoprecipitation

Cells were collected and treated with lysis buffer (150 mM NaCl, 50 mM Tris-Cl, 1% Triton X-100, protease inhibitor cocktail, pH 7.4). Supernatants were collected by centrifugation (15,000*g*, 15 min, 4 °C), and incubated with the indicated antibodies (1 μg ml^−1^) for 6 h at 4 °C, followed by immunoprecipitation with 20 μl protein A/G conjugated agarose (Santa Cruz Biotechnology). The precipitates were completely washed with lysis buffer and detected through immunoblotting.

### Immunofluorescence

Immunostaining was performed as described previously^57^,^58^. Briefly, cells were plated on 0.01% poly-L-Lysine-treated coverslips and fixed with 4% paraformaldehyde for 10 min, followed by permeabilization with 0.5% Triton X-100 for 20 min at room temperature. Primary antibodies (0.2 μg ml^−1^) were added for 2 h at room temperature post blocking with 10% donkey serum for 30 min. Samples were further stained with Alexa-488-, Alexa-594- or Alexa-405-conjugated secondary antibodies, followed by visualization with confocal microscopy (Olympus FV1000).

### RNA interference

RNA interference sequences were designed according to pSUPER system instructions (Oligoengine). BMDMs were electroporated with pSUPER-GFP vector encoding target sequences against PCBP2 (#1: 5′-GGTGCACGTATCAACATCT-3′, #2: 5′-GACCGACTAATGCCATCTT-3′); Ubc9 (#1: 5′-GGCACAATGAACCTGATGA-3′, #2: 5′-AGCAGAGGCCTACACAATT-3′); MAVS (#1: 5′-CAGAGAGCATCAAGAGCAA-3′, #2: 5′-GTCACAGTATCAGCCCTAT-3′); RIG-I (#1: 5′-CGCTAACCAAATTCCTGTC-3′, #2: 5′-GGTACAACATTGCGAGCAT-3′), and scramble sequences for 48 h, followed by GFP-positive cell selection through flow cytometry.

### Reverse transcription–PCR analysis

Total RNA was extracted from cells using Trizol reagent and cDNA was reverse transcribed using Superscript II (Invitrogen). Reverse transcription–PCR (RT–PCR) was performed using StarScript II Two-step RT–PCR Kit (Genestar) with the following primers: *Ifna* primers, sense: 5′-ACTCATAACCTCAGGAACAAG-3′, anti-sense: 5′-CTTTGATGTGAAGATG TTCAG-3′; *Ifnb* primers, sense: 5′-AGTACAACAGCTACGCCTGG-3′, anti-sense: 5'-GAGT CCGCCTCTGATGCTTA-3′; *Mavs*, sense: 5′-GCGAGGTCCACTGAGCTATC-3′, anti-sense: 5′-CAGGTCAGGAGCAATGGAGG-3′; *Pcbp2*, sense: 5′-TGGATGCCACAG TGACTTACG-3′, anti-sense: 5′-GGGAGGTGATTGAGGGCAAA-3′. VSV RNA was detected as described^59^.

### *In vitro* sumoylation reconstitution assay

cDNAs of SAE1, SAE2, Ubc9, SUMO2, PCBP2 and IRTKS were subcloned into pET-28a vectors. Plasmids were transformed into *E. coli* strain BL21 (DE3). DE3 clones were cultured (OD_600_=0.6), followed by induction with 0.2 mM isopropyl-β-D-thiogalactopyranoside at 16 °C for 24 h. Cells were collected and lysed by supersonic and further purified through Ni-NTA resin columns. The *in vitro* sumoylation reconstitution assays were performed by mixing SAE1/SAE2 (2 μg μl^−1^), Ubc9 (1.5 μg μl^−1^), SUMO2 (1 μg μl^−1^) and other proteins in the SUMO buffer (50 mMTris-HCl, 5 mM MgCl_2_, pH 8.0) to a volume of 18 μl. The reaction was started by adding 2 μl ATP buffer (2 mM ATP, 10 mM creatine phosphate disodium salt, 3.5 U ml^−1^ creatine kinase, 0.6 U ml^−1^ inorganic pyrophosphatase) at 37 °C for 2 h.

### Gene knockout in BMDMs through CRISPR-Cas9 technology

Genome engineering of the indicated genes was performed using the CRISPR-Cas9 system as described^60^. sgRNA upstream of PCBP2 exon 2: 5′-ACCATTACCAGTGG AATGGT-3′, sgRNA downstream of PCBP2 exon 2: 5′-CTTTTCTAGTGGAGTGTGGT-3′. WT BMDMs were infected with lentivirus to introduce the GFP-containing CRISPR/Cas9 vectors. GFP^high^ cells were sorted through flow cytometry, followed by analysis of editing efficiency through PCR. PCR products were subcloned and genotyped by DNA sequencing. Genotyped cells were transplanted to recipient mice for further experiments.

### Statistical analysis

Statistical significance was calculated by the indicated test as described in figure legends by GraphPad Prism 5.

## Additional information

**How to cite this article:** Xia, P. *et al*. IRTKS negatively regulates antiviral immunity through PCBP2 sumoylation-mediated MAVS degradation. *Nat. Commun.* 6:8132 doi: 10.1038/ncomms9132 (2015).

## Supplementary Material

Supplementary Information Supplementary Figures 1-13
