# Supplementary material for: IRTKS negatively regulates antiviral immunity through PCBP2 sumoylation-mediated MAVS degradation
Source: Nat Commun. 2015 Sep 8;6:8132. doi: 10.1038/ncomms9132 (PMC4569712; doi:10.1038/ncomms9132)
Supplement: Supplementary Information — Supplementary Figures 1-13 [file ncomms9132-s1.pdf]

Supplementary Figure 1

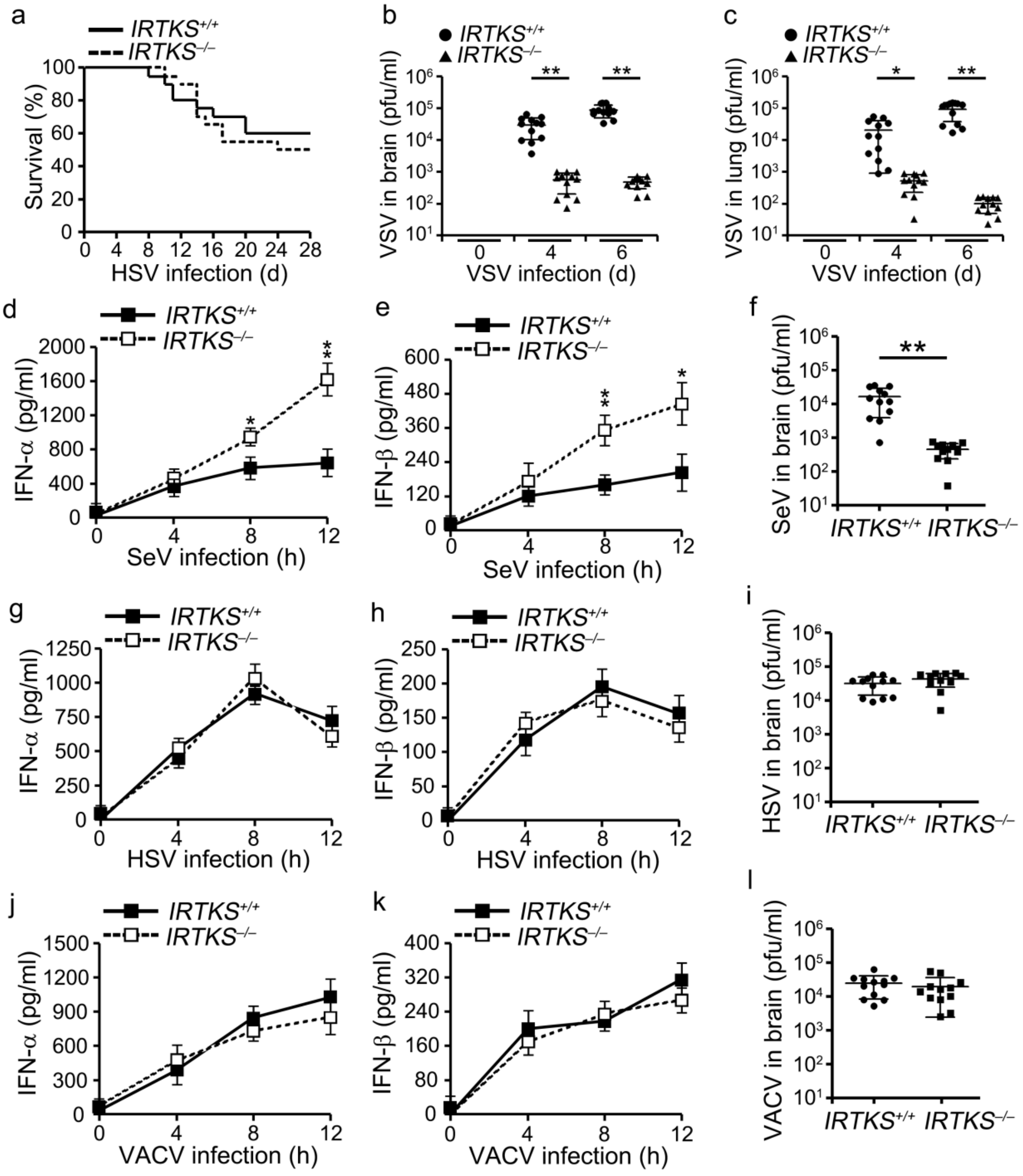

Supplementary Figure 1. *IRTKS*$^{-/-}$ mice exhibit elevated antiviral activity against RNA viruses, but not DNA viruses. (a) *IRTKS*$^{+/+}$ and *IRTKS*$^{-/-}$ mice were intranasally inoculated with HSV (5×10^5 pfu for each mouse). Survival curves were calculated. n=20. (b) *IRTKS*$^{+/+}$ and *IRTKS*$^{-/-}$ mice were intranasally inoculated with VSV (5×10^5 pfu for each mouse), followed by brain homogenization on the indicated days. Viral titers were examined through a plaque assay. n=10. (c) Viral load in the lung of *IRTKS*$^{-/-}$ mice. Lungs from mice treated as above were homogenized at the indicated times, followed by a plaque assay. n=12. (d, e) *IRTKS*$^{+/+}$ and *IRTKS*$^{-/-}$ mice were intranasally inoculated with Sendai virus (SeV) (1×10^6 pfu for each mouse), followed by detection of serum interferons by ELISA. n=12. (f) Viral load in the brain of *IRTKS*$^{-/-}$ mice. Brains from mice intranasally inoculated with SeV (1×10^6 pfu for each mouse) were homogenized on day 8, followed by viral titer examination. n=12. (g, h) *IRTKS*$^{+/+}$ and *IRTKS*$^{-/-}$ mice were intravenously

injected with HSV (1×10^6 pfu for each mouse), followed by analysis of serum interferons. n=12. (i) Brains from mice intravenously injected with HSV (1×10^6 pfu for each mouse) were homogenized on day 8, followed by viral titer examination. n=12. (j, k) *IRTKS*$^{+/+}$ and *IRTKS*$^{-/-}$ mice were intranasally inoculated with VACV (1×10^6 pfu for each mouse), followed by detection of serum interferons. n=12. (l) Brains from mice intranasally inoculated with VACV (1×10^6 pfu for each mouse) were homogenized on day 8, followed by viral titer examination. n=12. Data are shown as means±SD. A two-way ANOVA post hoc Bonferroni test was used. *, $P<0.05$; **, $P<0.01$. Data are representative of at least three independent experiments.

Supplementary Figure 2

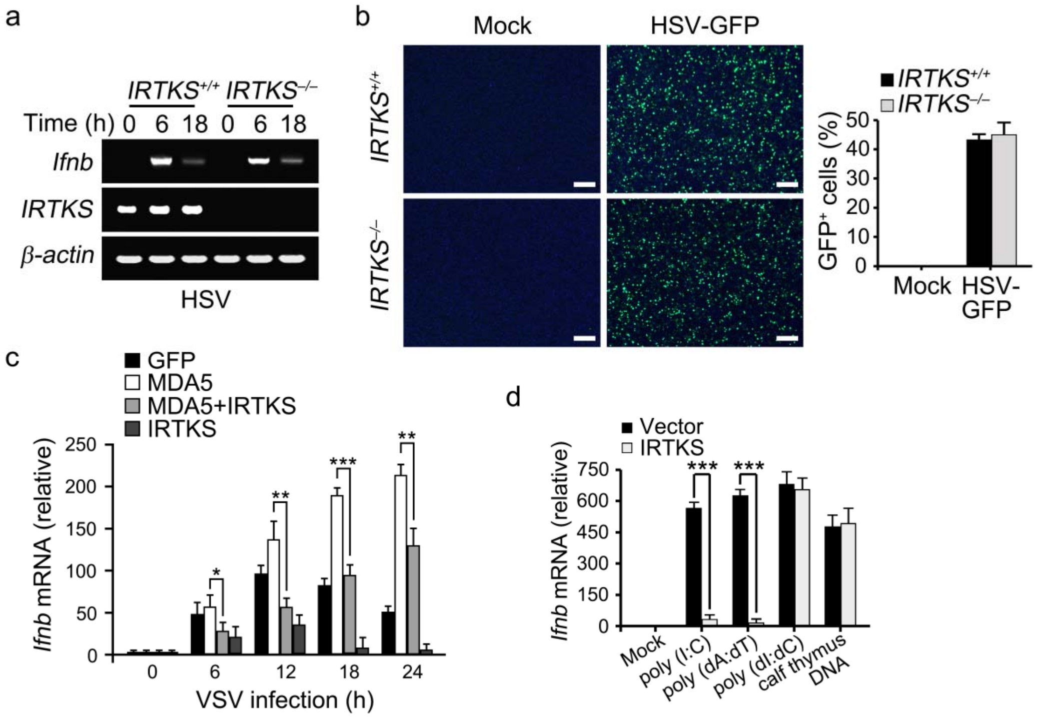

Supplementary Figure 2. IRTKS is distinctively involved in the innate immune responses against exogenous nucleic acids. (a) *IRTKS*$^{+/+}$ and *IRTKS*$^{-/-}$ BMDMs were infected with HSV (MOI=5) for the indicated times, followed by RNA extraction and RT-PCR analysis. (b) *IRTKS*$^{+/+}$ and *IRTKS*$^{-/-}$ BMDMs were infected with HSV-GFP (MOI=5) for 24 h, followed by examination with confocal microscopy (left panel). Cells were counterstained with DAPI. GFP positive cells were calculated (right panel). Scale bar, 200 μm. (c) WT MEFs were transfected with the indicated plasmids for 24 h, followed by infection with VSV (MOI=5) for the indicated times. *Ifnb* mRNA was analyzed by RT-PCR. (d) WT BMDMs expressing control vector or IRTKS were transfected with the indicated types of DNA or RNA (1 mg/ml) for 18 h, followed by detection of *Ifnb* mRNA through RT-PCR (normalized to β-actin). Data are shown as means±SD. A two-tail unpaired Student's *t*-test was used. ∗, $P<0.05$; ∗∗∗, $P<0.001$. Data were repeated at least three times with similar results.

Supplementary Figure 3

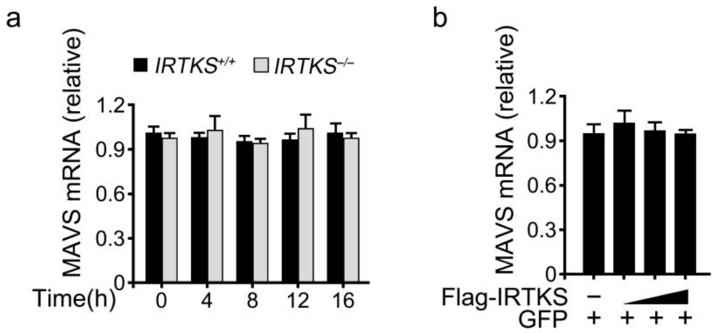

Supplementary Figure 3. IRTKS does not affect mRNA expression levels of MAVS. (a) *IRTKS*$^{+/+}$ and *IRTKS*$^{-/-}$ BMDMs were incubated with VSV (MOI=5) for the indicated times in the presence of 20 μg/ml CHX, followed by RNA extraction and RT-PCR analysis for MAVS mRNA. (b) Increasing amounts of Flag-tagged IRTKS and GFP control vector were co-transfected into BMDMs for 24 h, followed by RNA extraction and RT-PCR analysis. For (a), a two-tail unpaired Student's *t*-test was used; for (b), a one-way ANOVA followed by a Dunnett post hoc test was used using *PCBP2*$^{+/+}$ cells as controls. Data are representative of at least four independent experiments.

Supplementary Figure 4

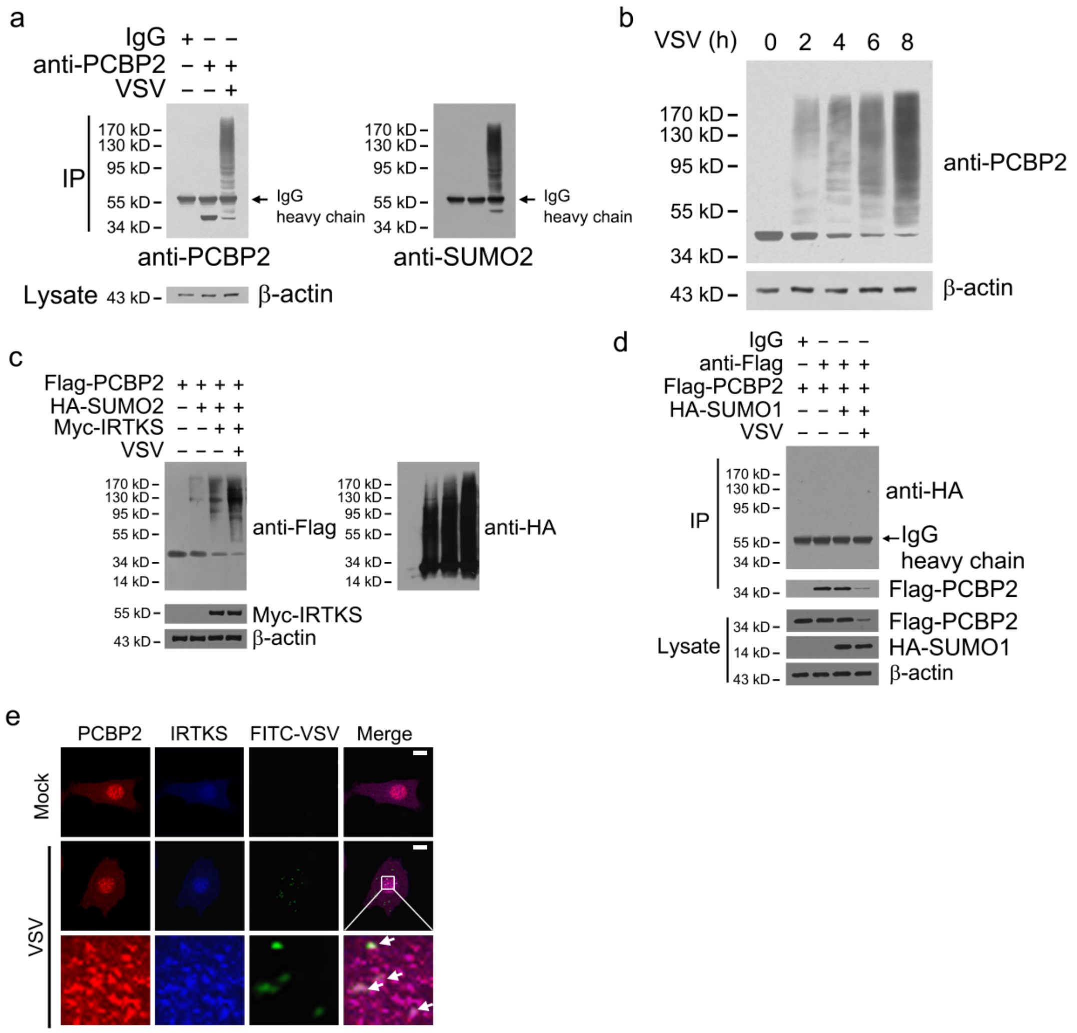

Supplementary Figure 4. PCBP2 undergoes polysumoylation post VSV infection. (a) WT BMDMs were incubated with VSV (MOI=5) for 8 h, followed by immunoprecipitation with anti-PCBP2 antibody. Immunoprecipitates were detected with anti-PCBP2 (left panel) or anti-SUMO2 antibody (right panel). (b) WT BMDMs were incubated with VSV (MOI=5) for the indicated times, whole cell lysates were probed with anti-PCBP2 antibody. β-actin was as a loading control. (c) WT MEFs transfected with the indicated plasmids were infected with VSV (MOI=5) for 8 h, whole cell lysates were immunoblotted with the indicated antibodies. (d) WT MEFs transfected with the indicated plasmids were infected with VSV (MOI=5) for 8 h, followed by immunoprecipitation with anti-Flag antibody. Immunoprecipitates were probed with the indicated antibodies. (e) WT BMDMs were incubated with FITC-conjugated VSV (MOI=5) for 1 h, followed by immunostaining with the indicated antibodies. Scale bar, 10 μm.

Supplementary Figure 5

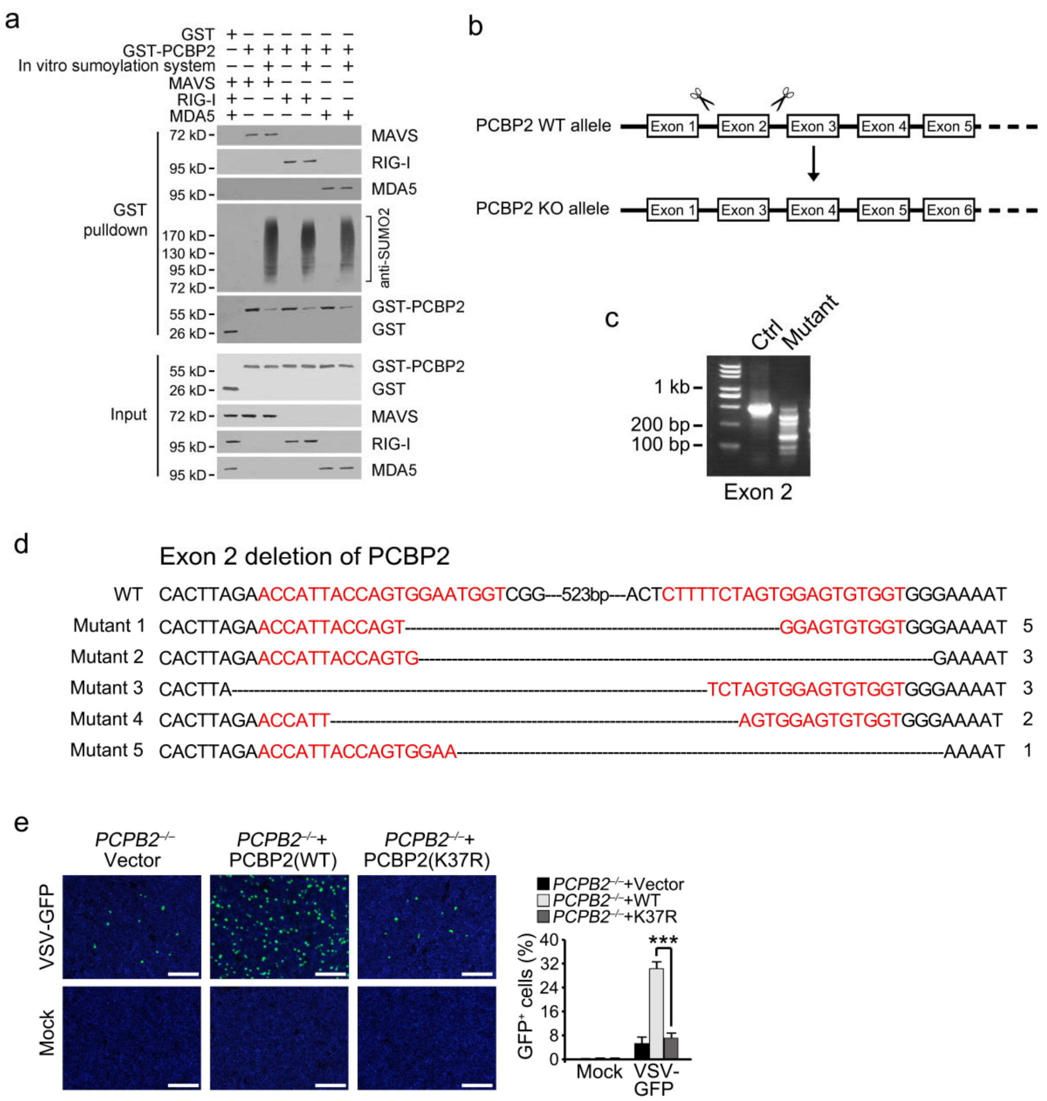

Supplementary Figure 5. PCBP2 sumoylation does not affect its interaction with MAVS, RIG-I or MAD5 and generation of PCBP2 knockout macrophages through a CRISPR/Cas9 approach. (a) PCBP2 sumoylation does not affect its association with MAVS, RIG-I or MDA5. GST or GST-PCBP2 was sumoylated in vitro and then incubated with recombinant MAVS, RIG-I or MDA5, followed by GST pulldown assays. Precipitates were immunoblotted with the indicated antibodies. (b, c) WT BMDMs were infected with CRISPR/Cas9-GFP lentivirus containing sgRNAs flanking exon 2 of PCBP2for 3 days, followed by flow cytometry-based sorting of GFP^{high} cells (b). Genomic editing efficiency was determined by the Surveyor assay (c). (d) PCR products containing the target regions were cloned and fourteen randomly selected clones were genotyped by DNA sequencing. The mutant alleles are shown and the numbers of the mutant alleles are listed in the right. (e) *PCBP2*$^{+/+}$ and *PCBP2*$^{-/-}$ BMDMs were rescued with WT or K37R-PCBP2, followed by infection with VSV-GFP (MOI=5) for 24 h. Cells were counterstained with DAPI (left panel). GFP positive cells were calculated (right panel). Scale bar, 200 μm. Data are shown as means±SD. A two-tail unpaired Student's *t*-test was used. Experiments were repeated at

least three times with similar results.

Supplementary Figure 6

Figure 1a anti-IRTKS

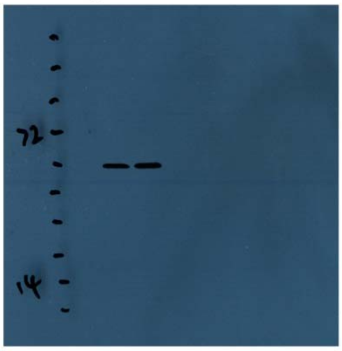

Figure 1a anti-β-actin

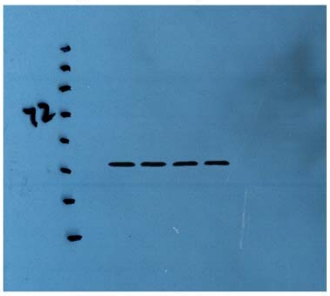

Supplementary Figure 6. Original blots in Figure 1.

Supplementary Figure 7

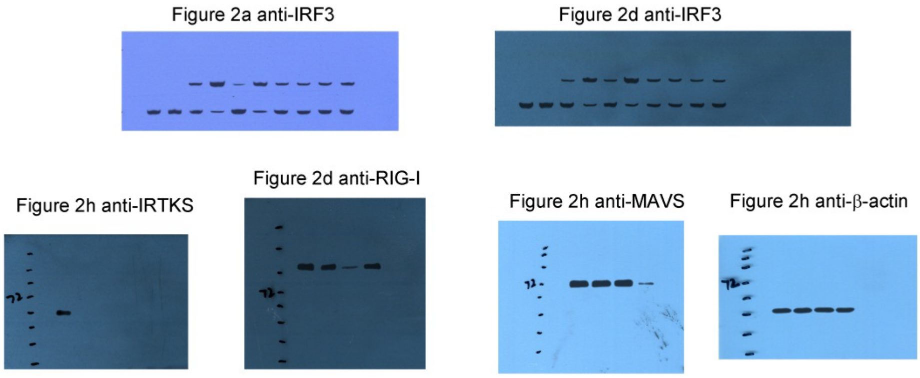

Supplementary Figure 7. Original blots in Figure 2.

Supplementary Figure 8

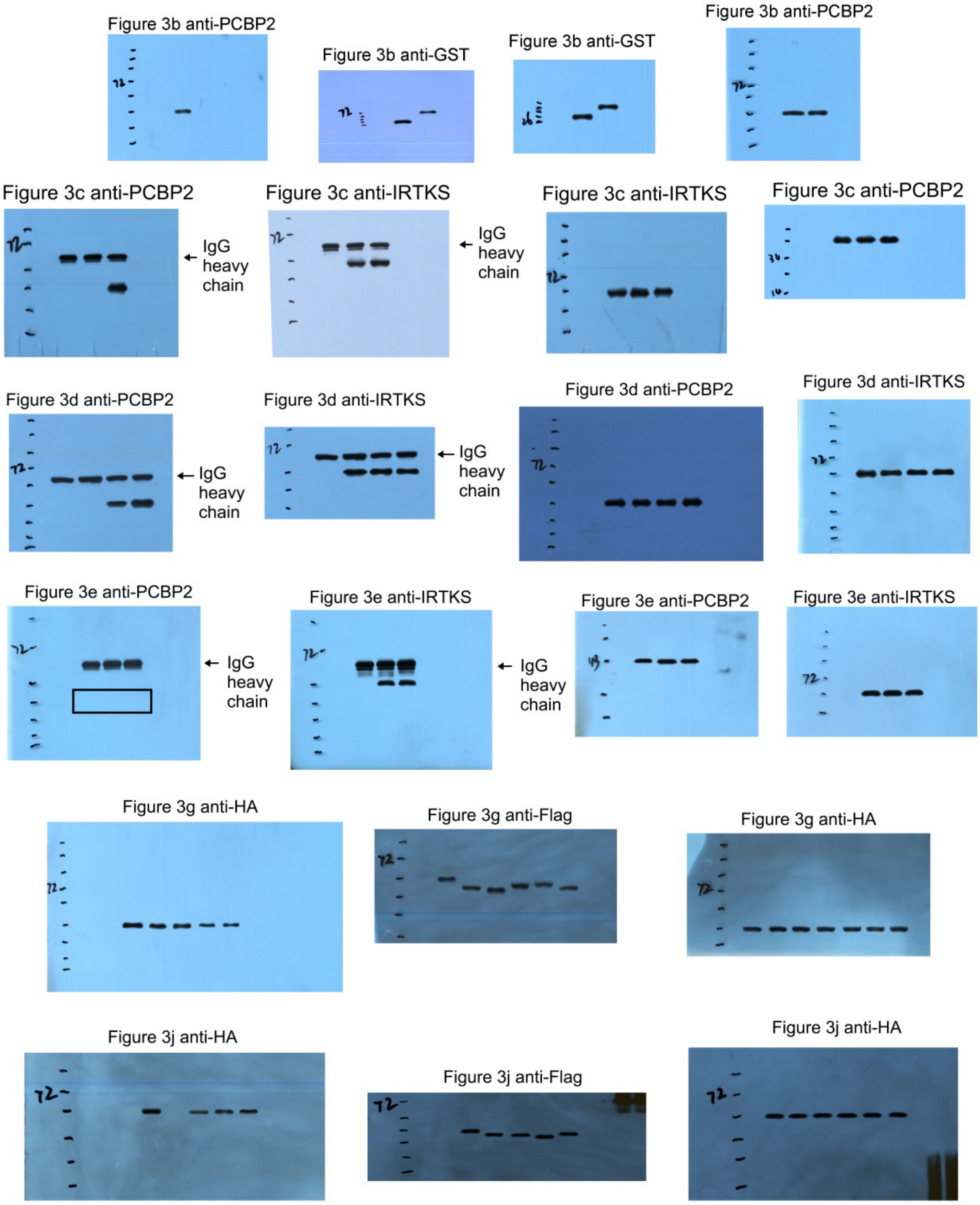

Supplementary Figure 8. Original blots in Figure 3.

Supplementary Figure 9

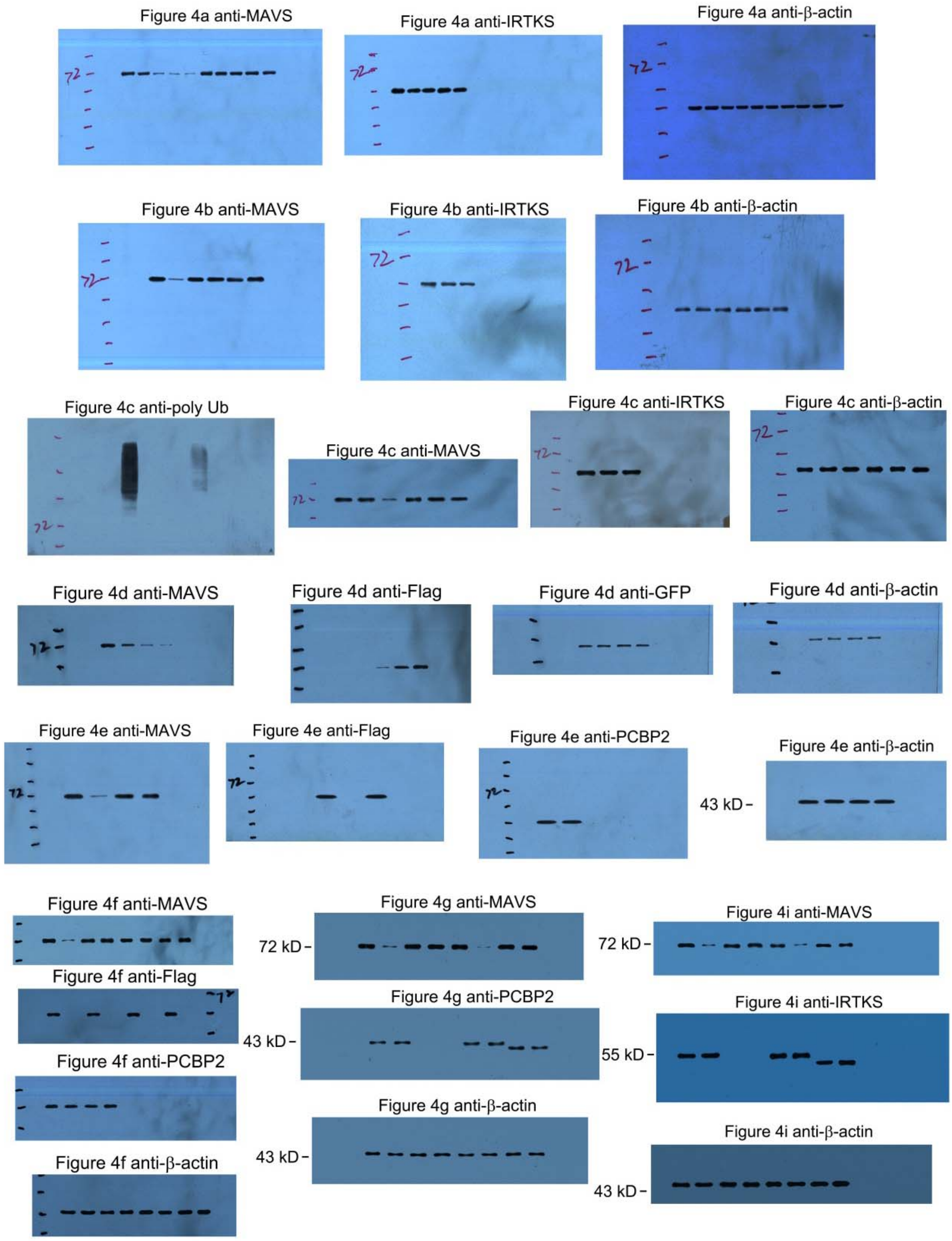

Supplementary Figure 9. Original blots in Figure 4.

Supplementary Figure 10

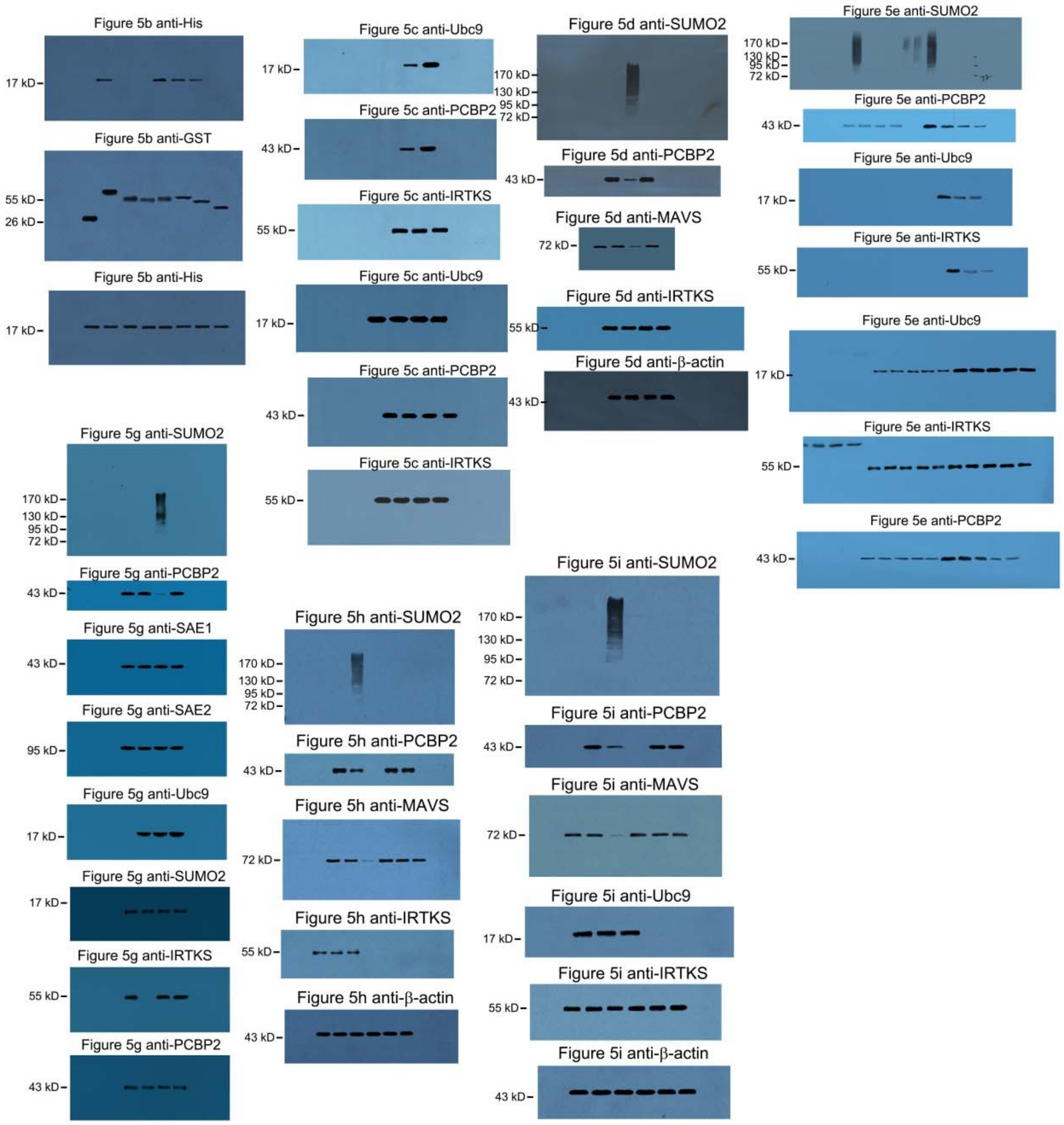

Supplementary Figure 10. Original blots in Figure 5.

Supplementary Figure 11

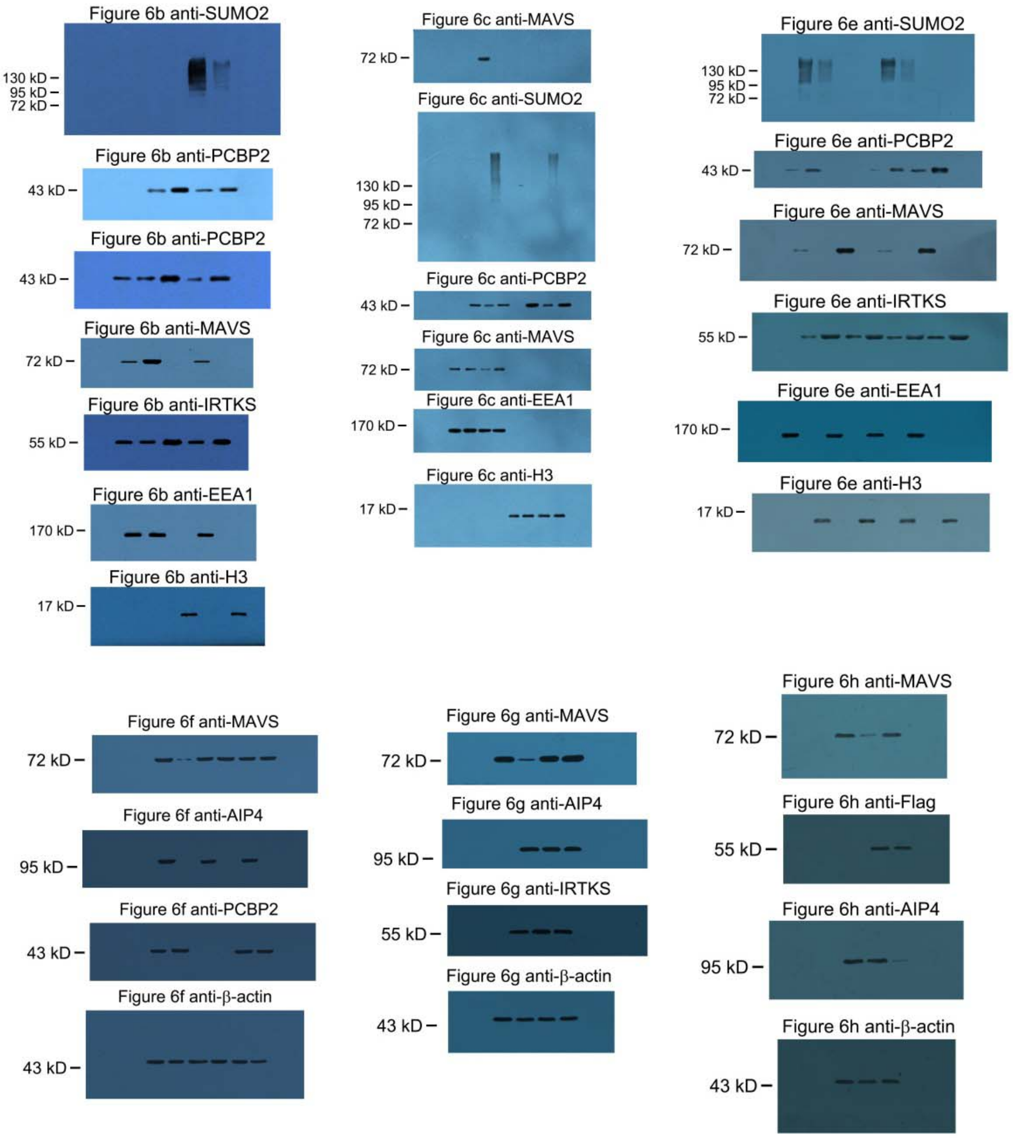

Supplementary Figure 11. Original blots in Figure 6.

Supplementary Figure 12

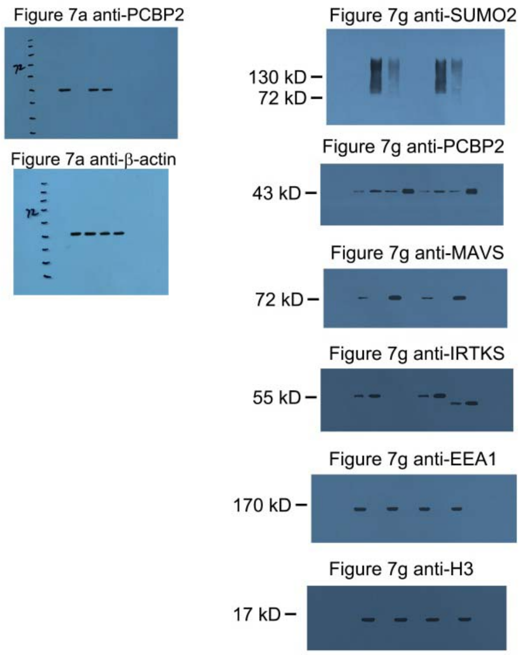

Supplementary Figure 12. Original blots in Figure 7.

Supplementary Figure 13

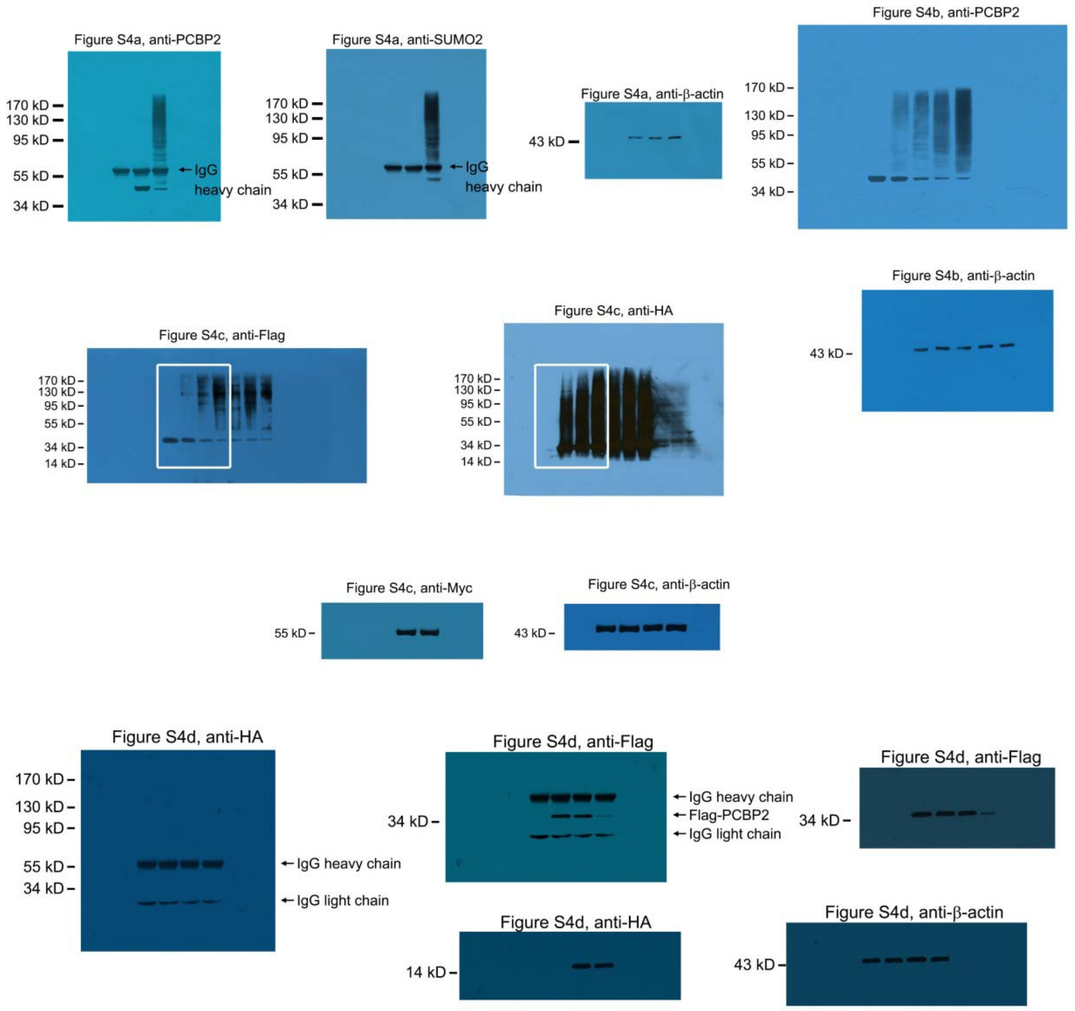

Supplementary Figure 13. Original blots in Supplementary Figure 4.
